# Macrophage polarization in disease therapy: insights from astragaloside IV and cycloastragenol

**DOI:** 10.3389/fphar.2025.1598022

**Published:** 2025-05-23

**Authors:** Bei-Bei Xiong, Yu-Mei Zhuo, Huan Wang, Qiao-Ling Zheng, Feng Tang, Qun Huang, Man Yao

**Affiliations:** ^1^ Department of Oncology, The First People’s Hospital of Shuangliu District, Chengdu, China; ^2^ School of Pharmacy, Chengdu University of Traditional Chinese Medicine, Chengdu, China; ^3^ Department of Respiratory and Critical Care Medicine, The First People’s Hospital of Shuangliu District, Chengdu, China; ^4^ Sichuan Academy of Medical Sciences, Sichuan Provincial People’s Hospital, Chengdu, Sichuan, China; ^5^ Department of Ophthalmology, Hospital of Chengdu University of Traditional Chinese Medicine, Chengdu, China; ^6^ Department of Ophthalmology, Chengdu First People’s Hospital, Chengdu, China

**Keywords:** astragaloside IV, cycloastragenol, macrophage polarization, nano drug delivery, natural products

## Abstract

Dysregulated activation and polarization of macrophages drive the pathogenesis of diverse diseases, including inflammatory, autoimmune, ischemic, metabolic disorders, and cancers. Despite therapeutic advances, precise regulation of macrophage polarization remains challenging. Natural products have recently emerged as promising therapeutic regulators. Astragaloside IV (AS-IV) and its hydrolysate cycloastragenol (CAG), which are bioactive compounds derived from *Astragalus membranaceus*, have garnered significant interest due to their notable pharmacological properties encompassing anti-inflammatory, immunomodulatory, and antitumor effects. Nevertheless, the intricate multi-pathway mechanisms through which AS-IV and CAG regulate macrophage polarization are still not fully understood. A systematic review of literature from PubMed, Google Scholar, and SciFinder (2013–2025) shows that AS-IV and CAG modulate macrophage polarization. These compounds target critical signaling pathways, including TLR4/NF-κB, PI3K-AKT, AMPK, and PPARγ. These compounds exhibit therapeutic potential by suppressing pro-inflammatory M1 phenotypes and promoting anti-inflammatory/reparative M2 phenotypes. Their activities include anti-inflammatory, tissue-regenerative, and antitumor effects, with applications in inflammatory diseases, autoimmune disorders, ischemic vascular pathologies, metabolic syndromes, and cancer therapy. Furthermore, the integration of nanotechnology has emerged as a transformative approach to significantly enhance the bioavailability and targeted delivery of AS-IV and CAG, thereby expanding their clinical applicability. Despite the significant therapeutic potential of AS-IV and CAG in various disease models, their clinical translation remains constrained by low bioavailability. Future advancements that incorporate gene-editing technologies, computer-aided drug design, and nanotechnology are anticipated to optimize their pharmacokinetics and clinical efficacy. These innovations may position AS-IV and CAG as transformative agents in future therapies.

## Graphical Abstract

**Figure:**
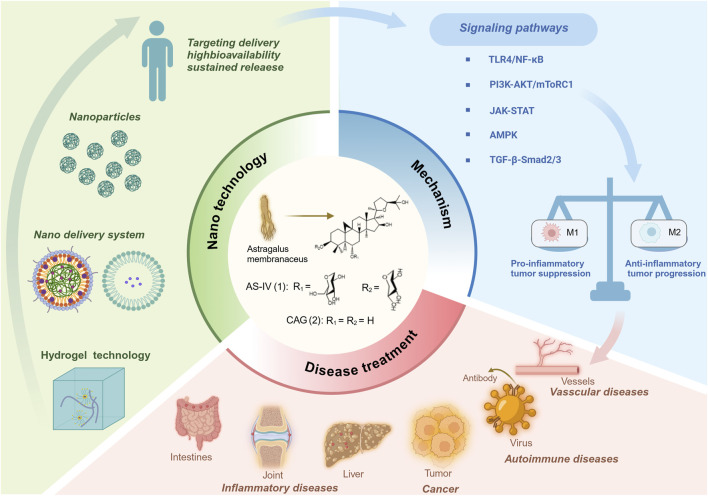
Astragaloside IV and cycloastragenol mitigate various diseases by modulating macrophage polarization.

## 1 Introduction

Astragaloside IV (AS-IV), a triterpenoid saponin derived from *Astragalus membranaceus* (Huangqi) (Wang S. et al., 2009). As the principal active components of *Astragalus*, AS-IV and its aglycone cycloastragenol (CAG) exhibit extensive biological activity and therapeutic potential, particularly in inflammatory and neoplastic disorders (Zhou et al., 2012). AS-IV and CAG also exert diverse pharmacological effects, including immune regulation, organ protection, hypoglycemic action, apoptosis modulation, and antiviral activity. Recent studies reveal that these compounds modulate key signaling pathways, including protein kinase B (Akt), mammalian target of rapamycin complex 1 (mTORC1), the Janus kinase-signal transducer and activator of transcription (JAK-STAT), NOD-like receptor family pyrin domain containing 3 (NLRP3) inflammasome, and peroxisome proliferator-activated receptor (PPAR)γ, thereby influencing macrophage polarization. For instance, AS-IV mitigates sepsis by inhibiting macrophage activation and polarization (Liu et al., 2016; Yang et al., 2024), while CAG alleviates neuroinflammation in Parkinson’s disease by promoting microglial autophagy and suppressing ROS-induced NLRP3 inflammasome activation (Feng et al., 2024). Additionally, both compounds show promise in addressing fibrosis-related diseases, highlighting their broad applicability and advancing our understanding of natural drug components (Wan et al., 2018).

Macrophages, as central players in inflammation, tissue repair, and tumor immunity, exhibit functional polarization states. The imbalance between pro-inflammatory (M1) and immunosuppressive (M2) macrophage polarization is mechanistically linked to tumor progression, chronic inflammation (such as rheumatoid arthritis) and metabolic diseases (such as atherosclerosis). M1 macrophages aggravate tissue damage by secreting TNF-α and IL-6, while M2 macrophages promote repair through IL-10 (Piccolo et al., 2017). Given the central role of macrophage polarization in disease progression, natural products like AS-IV and CAG have garnered attention for their multi-target regulation of macrophage polarization. For example, macrophages contribute to atherosclerosis by forming foam cells, while AS-IV mitigates this process by targeting the transforming growth factor β (TGF-β)-activated kinase (TAK) 1 signaling pathway, reducing macrophage adhesion and migration (Hua et al., 2025). In addition, CAG improves imiquimod-induced psoriasis-like inflammation in mice by selectively inhibiting NLRP3 inflammasome-mediated pyroptosis (Deng et al., 2019). These findings underscore the need to elucidate AS-IV and CAG mechanisms in macrophage polarization regulation, which could advance novel therapeutic strategies.

Despite their significant therapeutic potential, most studies on AS-IV and CAG focus on some of its mechanisms in specific diseases, lacking a comprehensive analysis of the regulatory mechanism on macrophage polarization, especially its potential role in a variety of diseases. There is also a lack of comprehensive analysis of the regulatory mechanism of macrophages in various diseases. In particular, research on their role in macrophage polarization remains limited. Recent studies on myocardial fibrosis have emphasized the ability of AS-IV to inhibit collagen deposition by regulating TGF-β/Smad signaling and oxidative stress pathways (Ren et al., 2023). It mainly focuses on the synergistic effect of compound preparations through the RAAS/NADPH oxidase axis synergistic anti-fibrosis-specific pathway. However, this study explores how AS-IV and CAG regulate macrophage polarization and their therapeutic applications in inflammatory, autoimmune, ischemic, metabolic diseases, and cancers. By dissecting their multi-pathway regulatory effects, this study not only highlights their therapeutic versatility but also provides a theoretical framework for developing macrophage-based treatment strategies. Furthermore, this study also summarizes the application of nanotechnology in the development of AS-IV and CAG drugs, which not only improves their bioavailability and addresses limitations related to low water solubility and bioavailability, but also offers innovative solutions for clinical applications. Finally, combined with gene-editing and computer-aided drug design (CADD), the prospect of its clinical transformation was discussed. Collectively, this review provides a fresh perspective on the modern medical applications of AS-IV and its hydrolysates, laying a robust foundation for future research and clinical practice.

## 2 Biosynthesis and metabolism of AS-IV and CAG

### 2.1 Biosynthetic pathways of AS-IV

#### 2.1.1 Enzymatic cascades in triterpenoid saponin synthesis

AS-IV is a triterpenoid saponin with a core structure composed of a tetracyclic triterpenoid skeleton and a glucose unit linked by a glycosidic bond. The unique balance of hydrophilicity, derived from multiple hydroxyls and glycosyl groups, and hydrophobicity, due to its tetracyclic structure, enables AS-IV to interact selectively with numerous biological targets, mediating its pharmacological properties (Magedans et al., 2021), such as antioxidant, anti-inflammatory, and immunomodulatory effects (Huang et al., 2014; Li L. et al., 2024; Zhong et al., 2022). AS-IV is predominantly sourced from the dry roots of *Astragalus membranaceus* or *Astragalus membranaceus* var. *mongholicus*, species found primarily in Northeast, North, and Northwest China, as well as Mongolia and South Korea (Fu et al., 2014). Initially isolated from methanol extracts of *A. membranaceus* roots using reverse column chromatography, early extraction methods were hindered by low yields and complex procedures (Kitagawa et al., 1983). Additionally, its intricate stereochemical rings and multiple chiral centers have posed significant challenges to its chemical synthesis.

In response to the challenges associated with chemical synthesis, the rapid advancements in biotechnology have led researchers to investigate biosynthetic pathways for the efficient production of AS-IV. The primary pathway underlying the biosynthesis of astragalosides during secondary metabolism is the mevalonate pathway (Augustin et al., 2011), comprising three major stages: the formation of intermediate mevalonate; the synthesis of isopentenyl pyrophosphate and dimethylallyl diphosphate (Ding et al., 2008); and the cyclization of 2,3-oxidized squalene (OS) to generate the triterpenoid saponin carbon ring skeleton (Huang et al., 2007). This biosynthetic process is further refined through chemical modifications, such as oxidation, reduction, and acetylation, catalyzed by enzymes including cytochrome P450s (CYP450s) and glycosyltransferases. Key rate-limiting enzymes—such as acetyl-CoA acetyltransferase, 3-hydroxy-3-methylglutaryl coenzyme A reductase, squalene synthase, squalene epoxidase, and cycloalkanol synthase—play essential roles in facilitating these reactions (He et al., 2008; Huang et al., 2007).

With the discovery of a triterpenoid biosynthetic gene cluster (BGC) has provided a comprehensive understanding of the AS-IV biosynthetic pathway (Xu et al., 2024). This pathway is characterized by sequential and selective chemical reactions, including hydroxylation, epoxidation, and glycosylation, mediated by three CYP450s, one 2-oxoglutarate-dependent dioxygenase (AmOGD1), and two glycosyltransferases. Specifically, oxidized squalene cyclase (AmOSC3) catalyzes the initial formation of cycloalkanols from OS. Subsequent oxidation reactions include C-16 hydroxylation catalyzed by AmCYP88D25 to generate 16-hydroxycycloalkanols, C-6 hydroxylation catalyzed by AmCYP88D7, and C-24,25 epoxidation catalyzed by AmCYP71D756 to form 24,25-epoxy-6,16-hydroxycycloalkanols (Chen et al., 2023b; Duan et al., 2023; Kim et al., 2014). AmOGD1 catalyzes the hydroxylation of the C-20 position to produce 3,20-dihydroxycycloalkanol, whose 20,24-tetrahydrofuran ring is formed by the spontaneous attack of the 20,24-dihydroxy group on the 24,25-epoxide (Xu et al., 2024). The final glycosylation reactions, catalyzed by UGTs, involve 3-O-xylosylation by AmGT11 and 6-O-glucosylation by AmGT36, culminating in the production of AS-IV (Zhang M. et al., 2022). The roles of these biosynthetic genes have been verified through gene silencing and heterologous expression experiments, enabling the successful heterologous production of AS-IV in *Nicotiana benthamiana* plants (Xu et al., 2024).

#### 2.1.2 Biosynthesis pathway from CAG to AS-IV

**FIGURE 1:**
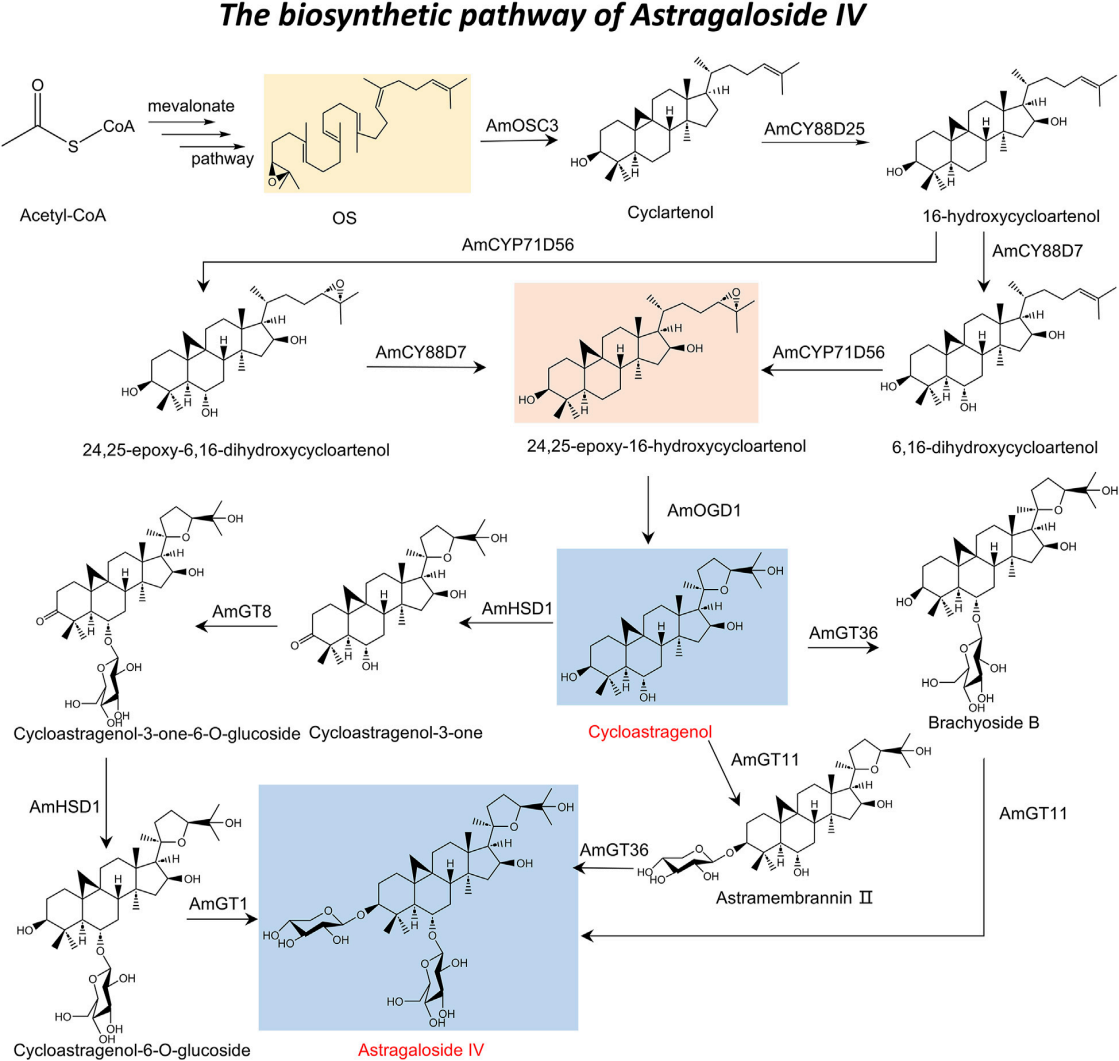
Total biosynthesis of astragaloside IV.

In addition, the biosynthetic pathway of AS-IV also encompasses its production from CAG through four critical steps (Zhang et al., 2024): C-3 oxidation, 6-O-glucosylation, C-3 reduction, and 3-O-xylosylation. This process is mediated by three key enzymes: AmHSD1, AmGT8, and AmGT1. AmHSD1 performs dual catalytic functions, facilitating both the C-3 oxidation of CAG and the C-3 reduction of cycloastragenol-3-one-6-O-glucoside, thereby demonstrating remarkable catalytic flexibility across diverse substrates, including pentacyclic triterpenes, tetracyclic triterpenes, and steroids. Additionally, AmGT8 and AmGT1 catalyze the 6-O-glucosylation and 3-O-xylosylation steps, respectively, which are essential for the final synthesis of AS-IV (Zhang M. et al., 2022; Chen et al., 2023a). Using transient expression experiments in tobacco (*Nicotiana tabacum*), researchers successfully simulated this biosynthetic process and achieved the production of AS-IV (Zhang et al., 2024). This study not only provides valuable insights into the intricate biosynthetic mechanisms of AS-IV but also lays a foundation for the efficient production of AS-IV by biotechnology. Drawing upon the aforementioned description, the complete biosynthetic pathway of AS-IV has been elucidated (Figure 1).

### 2.2 Bioconversion and metabolic fate of CAG

#### 2.2.1 Hydrolytic derivatization from AS-IV: microbial and enzymatic routes

CAG is primarily derived from the hydrolysis of AS-IV through various pathways, including Smith degradation, acid hydrolysis, microbial transformation, and enzymatic biotransformation (Wang and Chen, 2017; Li et al., 2019; Feng et al., 2014). While each method has its own advantages and limitations, with the development of biotechnology, enzymatic biotransformation has gained significant attention due to its high efficiency and environmental sustainability (Hegazy et al., 2015). Traditional methods, such as Smith degradation and acid hydrolysis, face several challenges. For instance, acid hydrolysis, which relies on the easy hydrolysis characteristics of the acetal or ketone structure of AS-IV under acidic conditions (e.g., HCl or H_2_SO_4_ hydrolysis), is hindered by harsh reaction conditions, high reagent costs, low yields, and the generation of numerous by-products (Feng et al., 2014). Similarly, Smith degradation, which involves selective oxidation of hydroxyl groups in AS-IV using periodate followed by borohydride reduction and specific hydrolysis under acidic conditions, is characterized by complex reaction protocols, expensive reagents, and low efficiency (Feng et al., 2014). In contrast, microbial transformation and enzymatic biotransformation offer higher selectivity and product purity. For example, *Bacillus* LG-502 can convert AS-IV to CAG with a conversion rate of up to 84% within 6 days (Wang and Chen, 2017). However, the practical application of microbial transformation is limited by the complexity of crude enzyme systems, which result in prolonged reaction times and relatively low selectivity for AS-IV conversion (Li et al., 2019).

To address these challenges, direct enzymatic biotransformation using purified enzymes has emerged as a more efficient and environmentally friendly approach. Recent studies have identified carbohydrate-induced β-glucosidase (Dth3) and β-xylosidase (Xln-DT) from *Thermotoga maritima* as highly selective enzymes capable of hydrolyzing the outer C-6 glucose and C-3 xylose of AS-IV, respectively (Li et al., 2019). Through a synergistic mechanism, Dth3 first hydrolyzes the C-6 glucose of AS-IV to produce the intermediate product Cyc B, which is subsequently hydrolyzed by Xln-DT at the C-3 xylose position to efficiently generate CAG. Under optimized reaction conditions (75°C, pH 5.5, 1 U of Dth3, and 0.2 U of Xln-DT), 1 g/L of AS-IV was converted to 0.63 g/L of CAG within 3 hours, achieving a molar conversion rate of 94.5%. Additionally, studies have also explored the role of intestinal bacteria in metabolizing AS-IV into CAG. For instance, lactic acid bacteria primarily produce CAG-2H by removing the C-6 glucose from AS-IV, whereas *Bifidobacterium* first removes the C-3 xylose to produce CAG (Takeuchi et al., 2022). These findings highlight the potential of gut microbiota in facilitating CAG synthesis. Future research leveraging metagenomics, culturomics, and other microbial omics techniques is expected to identify more efficient microorganisms and glycoside hydrolases, paving the way for the scalable and sustainable production of CAG.

#### 2.2.2 *In vivo* absorption, distribution, and hepatic metabolism

CAG, a tetracyclic triterpene compound with significant pharmacological activity, primarily functions as the aglycone of AS-IV *in vivo* (Rios and Waterman, 1997). The formation of CAG occurs predominantly through AS-IV metabolism, a process significantly influenced by intestinal activity, particularly the role of intestinal microorganisms (Zhu et al., 2010). In the gut, AS-IV undergoes deglycosylation by intestinal microbiota, converting it into secondary glycosides (cycloastragenol-6-glucoside) and aglycones (CAG) (Song et al., 2023). This metabolic process mainly takes place in the intestine, especially in intestinal feces. Following its formation, CAG is absorbed into intestinal epithelial cells via passive diffusion and subsequently subjected to first-pass metabolism in the liver (Zhu et al., 2010). In the intestine and liver, CAG undergoes various metabolic reactions, including deglycosylation, demethylation, hydroxylation, glucuronidation, sulfation, and cysteine binding, yielding a diverse array of metabolites (Yu et al., 2018). These metabolites enter systemic circulation, where they are distributed throughout the body to exert pharmacological effects (Ma et al., 2017). Studies on rat and human liver microsomes demonstrate that CAG is rapidly metabolized, with its metabolites exhibiting enhanced anti-inflammatory, antioxidant, and immunomodulatory activities, as well as broader systemic distribution (Song et al., 2023).

AS-IV and CAG have distinct but interconnected chemical structures and biosynthetic pathways. While AS-IV biosynthesis involves a series of complex enzymatic reactions and metabolic pathways, CAG production results from multiple hydrolysis pathways of AS-IV. These intricate biosynthetic and metabolic processes provide a robust theoretical foundation for elucidating the mechanisms underlying AS-IV and CAG’s roles in macrophage regulation.

## 3 Molecular mechanisms of AS-IV and CAG on macrophage polarization

### 3.1 Regulation of signaling pathways involved in macrophage polarization

#### 3.1.1 TLR4/NF-κB signaling pathway

Toll-like receptor 4 (TLR4) expressed on myeloid immune cells including macrophages, is a key pattern recognition receptor for pathogen-associated molecular patterns (Ciesielska et al., 2021). As the primary receptor for lipopolysaccharide (LPS), TLR4 mediates macrophage polarization through the TLR4/NF-κB signaling pathway. Upon LPS binding, TLR4 activates nuclear factor kappa B (NF-κB) through both MyD88-dependent and MyD88-independent pathways (Ciesielska et al., 2021; Rayees et al., 2020). Activated NF-κB translocates to the nucleus and binds promoter regions of pro-inflammatory genes, including tumor necrosis factor α (TNF-α), Interleukin (IL)-6, IL-1β, and inducible nitric oxide synthase (iNOS), thereby driving their expression (Shih et al., 2015). Elevated secretion of these pro-inflammatory cytokines is a hallmark of M1 macrophages, which exhibit strong inflammatory functions. In the MyD88-dependent pathway, TLR4 recognizes pathogen-associated molecular patterns like LPS, thereby activating its cytoplasmic TIR domain, which recruits the adaptor protein MyD88 (Deguine and Barton, 2014). MyD88 interacts with IL-1R-associated kinase (IRAK) 4 through its death domain, facilitating IRAK4-mediated phosphorylation and activation of IRAK 1 (Deguine and Barton, 2014). In turn, IRAK1 interacts with tumor necrosis factor receptor-associated factor (TRAF) 6 (Kollewe et al., 2004). TRAF6 engages ubiquitin-conjugating enzymes, such as UBC13 and UEV1A, catalyzing K63-linked polyubiquitination of key signaling proteins, including TRAF6, TAK1. This ubiquitination enables recruitment of TAK1-binding proteins 2 or TAK1-binding proteins 3, forming the TAK1 protein kinase complex (Walsh et al., 2015). Activation of this complex leads to phosphorylation of inhibitor of kappa B kinase α/β-NF-κB and mitogen-activated protein kinases, resulting in the nuclear translocation of NF-κB and upregulation of pro-inflammatory genes such as IL-1β and TNF-α (Walsh et al., 2015). This cascade shifts macrophage polarization toward the M1 phenotype, amplifying the pro-inflammatory functions. The MyD88-independent pathway, mediated by the TIR-domain-containing adaptor-inducing interferon-β, can also recruit TRAF6 or TRAF3. TRAF6 activation triggers TAK1 complex phosphorylation, converging on similar downstream mechanisms described in the MyD88-dependent pathway (Karnati et al., 2015).

While the TLR4/NF-κB pathway is essential for mounting effective immune responses, excessive or chronic activation can result in pathological outcomes (Sehnert et al., 2020). Overactivation of this signaling cascade drives hyperpolarization of macrophages to the M1 phenotype, leading to an overproduction of inflammatory factors that can cause tissue damage and contribute to the progression of numerous diseases. For example, in atherosclerosis, macrophages utilize TLR4 to recognize oxidized low-density lipoproteins, activating NF-κB and increasing inflammatory cytokine production. This exacerbates foam cell formation, plaque instability, and inflammatory responses, fueling disease progression (Huang et al., 2021). Moreover, NF-κB signaling extends beyond inflammation, as it regulates genes associated with cell cycle progression and apoptosis suppression, such as *c-Myc*, *cyclin D1*, *Bcl-2*, and *Bcl-xL* (Karin, 2006). Through these mechanisms, NF-κB also facilitates macrophage polarization to the M2 phenotype, effectively suppressing anti-tumor immune responses while promoting tumor growth and metastasis. These dual roles underscore the complexity of the TLR4/NF-κB signaling pathway in both immunity and disease pathogenesis.

#### 3.1.2 JAK-STAT signaling pathway

JAK-STAT signaling pathway plays a pivotal role in regulating macrophage polarization and function. Members of the STAT family, including STAT1, STAT3, and STAT6, are central to this process. Upon binding to its receptor, interferon-γ (IFN-γ) activates receptor-associated tyrosine kinases JAK1 and JAK2, which subsequently phosphorylate tyrosine residues (Tyr701) on STAT1 (Ivashkiv, 2018). These phosphorylated residues serve as docking sites for STAT proteins or other proteins containing SH2 domains. Phosphorylated STAT proteins dissociate from the receptor, dimerize via their SH2 domains, and translocate into the nucleus. Once in the nucleus, these dimers bind to gamma-activated sequence (GAS) elements in DNA, directly initiating the transcription of interferon-stimulated genes (O'Shea et al., 2015; Durham et al., 2019). These interferon-stimulated genes encode a range of immunologically active molecules, including chemokines, antigen-presenting molecules, phagocytic receptors, and factors with antiviral or antibacterial properties (Raftery and Stevenson, 2017). In addition to this direct transcriptional regulation, IFN-γ remodels the macrophage gene expression landscape through STAT1-driven transcription factors, such as interferon regulatory factors, as well as epigenetic mechanisms involving chromatin remodeling, enhancer activation, and gene-specific inhibition (Ivashkiv, 2018). These mechanisms collectively drive macrophage polarization toward the M1 phenotype, characterized by a pro-inflammatory profile.

Conversely, IL-4 induces JAK activation upon receptor binding, promoting the phosphorylation of STAT3 and STAT6. Phosphorylated STAT6 translocates to the nucleus to regulate the expression of M2 macrophage-associated genes, including arginase-1, mannose receptor type C-1, found in inflammatory zone protein 1, and Ym-1 (Litterst and Pfitzner, 2001). Furthermore, STAT6 alters chromatin structure and enhances gene transcription by recruiting co-activators such as CBP/p300, Steroid receptor coactivator 1, and poly (ADP-ribose) polymerase family member 14 (Litterst and Pfitzner, 2001). Activated STAT6 can also interact with transcription factors krüppel-like factor 4 and PPARγ to further amplify M2 macrophage polarization (Song et al., 2021).

Recent studies have highlighted the therapeutic potential of targeting this pathway. For instance, in the treatment of insulitis, chitosan oligosaccharides activate STAT6 by forming hydrogen bonds and salt bridge interactions within its active pocket. This activation promotes macrophage polarization toward the anti-inflammatory M2 phenotype, mitigating inflammatory responses (Leslie et al., 2019). Additionally, suppressors of cytokine signaling regulate JAK-STAT signaling by inhibiting JAK activity directly or interfering with STAT phosphorylation and receptor docking sites. These regulatory mechanisms serve as critical checkpoints to prevent excessive or inappropriate macrophage polarization, underscoring the importance of balanced JAK-STAT signaling in immune homeostasis and disease treatment (Dai et al., 2022).

#### 3.1.3 PI3K-Akt signaling pathway

The phosphatidylinositol 3-kinase (PI3K)-Akt signaling pathway plays a pivotal role in macrophage activation and polarization by modulating intracellular signaling cascades and metabolic processes. PI3K phosphorylates phosphatidylinositol 4,5-bisphosphate (PIP2) to generate phosphatidylinositol 3,4,5-trisphosphate (PIP3), which subsequently activates its primary downstream effector, Akt (Song et al., 2005). PI3K activation is triggered through receptor tyrosine kinases, growth factors, or cytokines such as IL-4 binding to their respective receptors (Luyendyk et al., 2008). This activation promotes the conversion of PIP2 into PIP3, which in turn facilitates phosphorylation of Akt at both Thr308 and Ser473 residues (Linton et al., 2019). Notably, phosphorylation at the Ser473 residue is essential for the maximal activation of Akt kinase. Experiment indicates that activation or overexpression of PI3K and Akt kinases suppresses LPS-induced macrophage activation. Conversely, non-specific chemical inhibition of PI3K signaling in TLR-activated cells enhances NF-κB activation and iNOS expression, thereby promoting M1 macrophage response (Covarrubias et al., 2015).

Beyond modulating M1 responses, activated Akt phosphorylates downstream targets, including the mechanistic target of rapamycin (mTOR). The mTOR pathway supports M2 macrophage polarization by enhancing protein synthesis, cell proliferation, intracellular nutrient sensing, and energy metabolism (Laplante and Sabatini, 2012). For instance, studies by Vergadi et al. demonstrate that Akt-driven activation of mTOR promotes M2 macrophage polarization, facilitating anti-inflammatory responses and tissue repair. In addition to its role in metabolism, Akt impacts transcription factor activity, such as that of STAT6 (Vergadi et al., 2017). Akt phosphorylates STAT6, enhancing its nuclear stability and transcriptional activity, thereby further promoting M2 macrophage polarization. Supporting this, research by Zhang et al. revealed that TGF-β drives M2-type polarization and upregulates IL-10 expression via the Akt/STAT6 signaling axis (Zhang et al., 2016). Furthermore, the PI3K/Akt1 pathway regulates macrophage polarization by inhibiting pro-inflammatory signals. Specifically, this pathway suppresses TRAF6 while regulating the TLR4 inhibitor IRAK-M. By inactivating the transcription factor forkhead box transcription factor O1(FoxO1), the PI3K/Akt1 pathway mitigates the expression of TLR4 target genes, thereby favoring the polarization of macrophages toward the anti-inflammatory M2 phenotype (Liu et al., 2019).

#### 3.1.4 AMPK signaling pathway

Adenosine monophosphate-activated protein kinase (AMPK) serves as a critical upstream regulator of anti-inflammatory signaling pathways, activated via classical (AMP/ADP-dependent) and non-classical (AMP/ADP-independent) mechanisms (Steinberg and Hardie, 2023; Russell and Hardie, 2020). AMPK activation mitigates inflammatory responses by inhibiting glycolysis, promoting oxidative phosphorylation, and shifting macrophages toward an anti-inflammatory phenotype. In terms of glucose metabolism, macrophage subsets exhibit distinct metabolic profiles: M1 macrophages rely heavily on glycolysis, whereas M2 macrophages predominantly depend on oxidative phosphorylation (Dzik, 2021). Upon activation, AMPK exerts dual regulatory effects on cellular metabolism. It enhances glycolytic activity by phosphorylating phosphofructokinase 2, facilitating the conversion of fructose-6-phosphate to fructose-1,6-bisphosphate, thus augmenting glycolysis in M1 macrophages. Concurrently, AMPK inhibits cholesterol biosynthesis and indirectly regulates fatty acid synthase activity, modulating lipid metabolism, and contributing to macrophage polarization (Szewczuk et al., 2020). Beyond its metabolic functions, AMPK intersects with the mTOR signaling pathway, indirectly influencing amino acid metabolism to maintain macrophage immune activity. As a pivotal upstream signal, AMPK initiates anti-inflammatory pathways by promoting IL-10 production and suppressing pro-inflammatory cytokine expression during macrophage polarization mediated by the PI3K/Akt/mTORC1 and STAT3 axes (Zhu et al., 2015). Notably, when macrophages are activated by IL-4 and IL-13, the phosphorylation of AMPK can enhance PPARδ and angiotensin-converting enzyme expression, driving M2 macrophage polarization (Zhou et al., 2014).

AMPK activation further dampens inflammation by reducing inhibitor of NF-κB degradation, thereby restricting NF-κB activation, while simultaneously enhancing Akt activity (Sag et al., 2008). This dual mechanism decreases M1 polarization while promoting the anti-inflammatory M2 phenotype. A notable example is the drug metformin, which inhibits cancer cell polarization to the M2 macrophage via the AMPK-NF-κB signaling pathway by downregulating M1-related cytokines and upregulating M2-related cytokines (Chiang et al., 2017). Metformin’s regulatory role highlights the broader significance of AMPK activation in inflammatory contexts. Moreover, AMPK restores cholesterol homeostasis in macrophages, inhibits foam cell formation, and reduces the accumulation of pro-inflammatory lipid intermediates, ultimately mitigating the progression of atherosclerosis (Wang et al., 2017).

#### 3.1.5 Other signaling pathways

In addition to the well-characterized pathways, macrophage polarization is influenced by several other signaling mechanisms that collectively determine their functional states. The PPARγ plays a crucial role in modulating macrophage polarization by integrating signals from diverse pathways. Upon activation, PPARγ can directly bind to the promoter regions of inflammation-associated genes, suppressing the release of pro-inflammatory mediators. This suppression occurs via the competitive inhibition of key transcription factors, such as NF-κB, activator protein-1, and STAT (Kundu et al., 2014). Notably, the interplay between PPARγ and NF-κB is critical for maintaining the balance between M1 and M2 macrophages. By inhibiting the JAK2/STAT1 pathway, PPARγ promotes M2 macrophage polarization (Luo et al., 2017). Conversely, TGF-β can facilitate M1 macrophage polarization through ubiquitination and degradation of PPARγ (Li X. et al., 2023). For instance, schisandrin B has been shown to bind PPARγ, activate it signaling pathway, attenuate NF-κB activation, reduce inflammation, and exert protective effects against liver fibrosis (Chen Q. S. et al., 2021).

FoxO1 acts as a downstream effector of the Akt signaling pathway and is intricately linked to macrophage polarization. TGF-β inhibits Akt signaling, thereby enhancing the nuclear translocation of FoxO1, which promotes M2 polarization while suppressing M1 polarization. Evidence suggests that TGF-β secreted by mesenchymal stem cells modulates macrophage polarization via the Akt/FoxO1 signaling pathway, downregulating M1-associated genes and upregulating M2 markers such as IL-10. This axis also increases macrophage phagocytic capacity, enhances pathogen clearance, and alleviates sepsis-related symptoms (Liu et al., 2019). Additionally, high mobility group protein B1 inhibits NF-κB p50 transcriptional activity, thereby deactivating the NF-κB signaling pathway and steering macrophage polarization toward the M2 phenotype (Liu et al., 2024).

**FIGURE 2:**
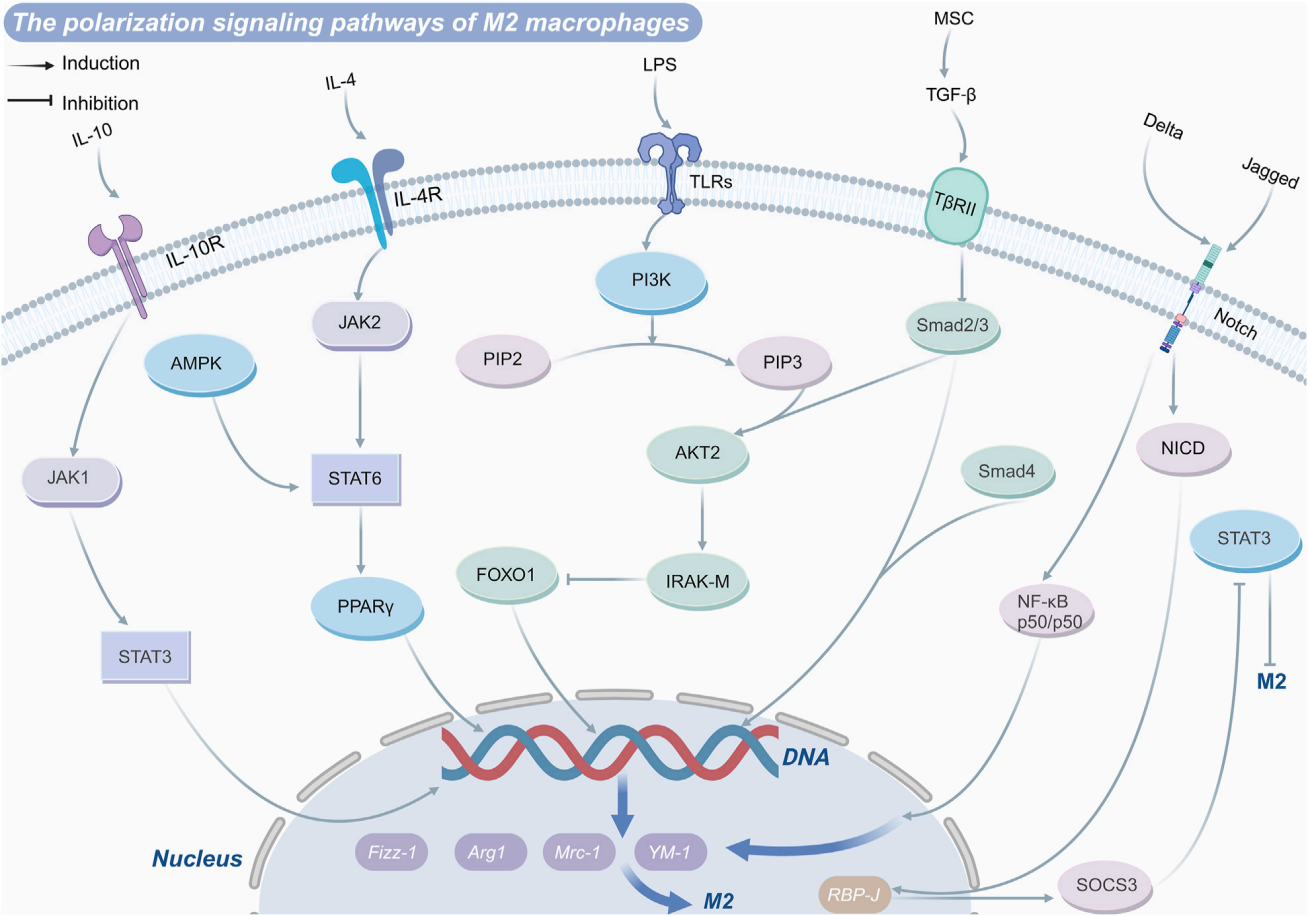
M2 polarization-regulating signaling pathways macrophage.

**FIGURE 3:**
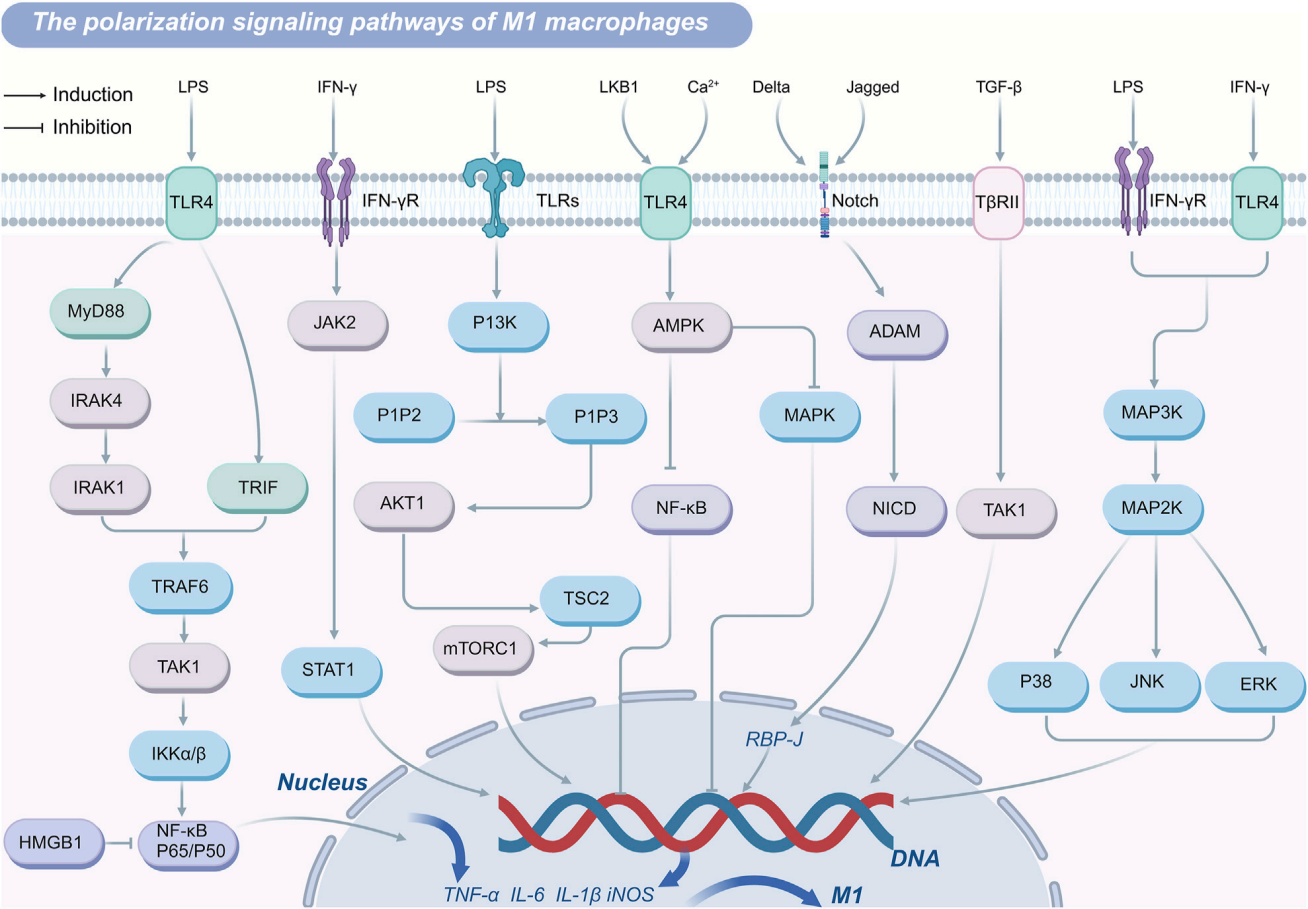
M1 polarization-regulating signaling pathways in macrophage.

Overall, there are various gene pathways induction of M2 macrophage polarization (Figure 2) and/or inhibition of M1 macrophage polarization (Figure 3) and drug targets. The TLR4/NF-κB axis plays a central role in recognizing pathogens and inducing the expression of pro-inflammatory genes to drive M1 polarization, though excessive activation can cause inflammatory damage. PPARγ and FoxO1 regulate the M1/M2 balance by curbing NF-κB activity and other pro-inflammatory pathways. Similarly, the JAK-STAT pathway modulates polarization, with IFN-γ driving M1 differentiation through STAT1 activation, and IL-4 promoting M2 polarization via STAT3 and STAT6. The PI3K-Akt signaling pathway supports M2 metabolism and function by activating mTOR while simultaneously suppressing TLR4 signaling to favor M2 polarization. The AMPK pathway facilitates M2 polarization by modulating metabolic pathways and suppressing inflammatory signals. These interconnected pathways collectively dictate macrophage polarization, shaping immune responses, tissue repair, and disease progression.

### 3.2 Types and functions of macrophage polarization

Macrophages, as highly plastic immune cells, play multifaceted roles in the onset and progression of various diseases. Their polarization states not only regulate inflammatory responses and facilitate tissue repair and regeneration but can also exacerbate tissue damage, drive pathological processes during infections and inflammatory diseases, and influence tumor growth, invasion, and metastasis. Upon tissue damage or infection, macrophages migrate from the bloodstream to affected tissues, where they are influenced by local growth factors, pro-inflammatory cytokines, and microbial products, leading to increased infiltration into inflammatory sites (Ingersoll et al., 2011; Nourshargh and Alon, 2014). Macrophage infiltration is a hallmark of chronic inflammation and cancer. Once recruited to the lesion, macrophages polarize into two primary subtypes in response to specific stimuli: classically activated M1 macrophages and alternatively activated M2 macrophages (Austermann et al., 2022). M1 macrophages, activated by LPS or T helper cell (Th) 1 cytokines, such as IFN-γ, produce pro-inflammatory cytokines, including TNF-α and IL-1β. These cytokines amplify inflammation, recruit additional immune cells to sites of infection or injury, and promote pathogen clearance. In contrast, M2 macrophages, activated by Th2 cytokines such as IL-4 and IL-13, secrete anti-inflammatory cytokines, including IL-10 and TGF-β, which promote wound healing, tissue reconstruction, and the resolution of inflammation (Martinez et al., 2008; Sica and Mantovani, 2012). For example, in rheumatoid arthritis, TNF-α produced by M1 macrophages stimulates synovial cells to release additional cytokines, perpetuating chronic polyarthritis (Xu et al., 2025). Similarly, macrophage polarization is implicated in the pathogenesis of chronic demyelinating diseases of the central nervous system (CNS) (Du et al., 2017). In the experimental autoimmune encephalomyelitis model, M1 macrophages recruited to the CNS promote Th1 effector responses, while myeloid cells producing IL-23 stimulate Th cells to secrete granulocyte-macrophage colony-stimulating factor, exacerbating disease severity. Conversely, M2 macrophages mitigate multiple sclerosis by inducing T cell apoptosis and secreting anti-inflammatory cytokines, such as TGF-β and IL-10, which terminate the inflammatory response. Recent studies have shown that blocking the C-C motif chemokine ligand (CCL) 5/CCL7-CCR1 axis reduces NF-κB pathway activation, limits M1 macrophage polarization, alleviates articular cartilage damage, and slows the progression of osteoarthritis (Jiang et al., 2014; Nakagawa and Chiba, 2015).

Macrophages also exhibit dual roles in cancer. M1 macrophages possess anti-tumor properties, effectively identifying and destroying cancer cells through phagocytosis and cytotoxicity (Bernsmeier et al., 2020). They also enhance cytotoxic T cell activity and antibody-dependent cell-mediated cytotoxicity, thereby inhibiting tumor growth (Sica and Bronte, 2007). However, M2 macrophages often promote tumor progression. Within the tumor microenvironment (TME), tumor-associated macrophages predominantly adopt an M2-like phenotype, secreting cytokines such as IL-10 and TGF-β, chemokines like CCL2 and CCL5, and proteases such as matrix metalloproteinases (MMPs) (Hao et al., 2017; Annamalai et al., 2018). These factors collectively enhance tumor angiogenesis, growth, metastasis, and immunosuppression. Chemokines, including CCL2, CCL5, and platelet-derived growth factor, facilitate the recruitment of M2 macrophages into the TME (Murdoch et al., 2004; Allavena et al., 2008). Clinically, increased M1 macrophage infiltration correlates with improved survival outcomes in patients with lung cancer (Sedighzadeh et al., 2021), colon cancer (Wang H. et al., 2021), ovarian cancer (Zhang et al., 2014), and breast cancer (Cao et al., 2021). Conversely, a higher density of M2 macrophages is associated with poor prognosis in cancers such as colon cancer (Guo et al., 2022), non-small cell lung cancer (Zheng et al., 2020), and hepatocellular carcinoma (HCC) (Wang J. et al., 2021). Recent research has revealed that exosomes derived from head and neck squamous cell carcinoma promote M1 macrophage polarization by modulating STAT1, NF-κB, and activator protein-1 signaling pathways, providing novel insights into the role of tumor-derived exosomes in shaping macrophage-mediated TME dynamics (Yadav et al., 2025). Additionally, studies on ginseng-derived compounds have shown that they can modulate the tumor microenvironment by regulating macrophage polarization and enhancing anti-tumor immunity (Li M. et al., 2021).

Macrophage autophagy also plays a critical role in regulating polarization and influencing their function in inflammation and fibrosis. As an intracellular degradation system, autophagy maintains cellular homeostasis by removing damaged organelles and protein aggregates (Sun et al., 2021). Impaired autophagy in macrophages skews polarization toward the pro-inflammatory M1 phenotype, exacerbating immune responses and contributing to chronic inflammation and tissue damage, as observed in the livers of obese mice (Liu et al., 2015). Conversely, the regulation of autophagy flux by ubiquitin-specific protease 19 promotes anti-inflammatory M2 polarization (Liu T. et al., 2021). Several small-molecule drugs have also been shown to induce M2 polarization by activating autophagy. For example, spermine, an autophagy inducer, inhibits M1 polarization of liver-resident macrophages (Kupffer cells) while promoting M2 polarization in livers treated with thioacetamide (Zhou et al., 2018). Similarly, macrophage autophagy plays a protective role in fibrotic diseases. Dioscin, for instance, reduces silica-induced mitochondrial reactive oxygen species accumulation by activating alveolar macrophage autophagy, downregulating mitochondrial-dependent apoptosis, and decreasing the secretion of inflammatory factors and chemokines, ultimately attenuating pulmonary fibrosis (Du et al., 2019).

In short, macrophages play a pivotal role in the pathogenesis of numerous diseases, with their polarization states (M1 pro-inflammatory and M2 anti-inflammatory) exerting profound effects on disease progression. Regulating macrophage polarization offers a promising strategy to modulate inflammatory responses, facilitate tissue repair, and reshape the TME. These findings provide a strong theoretical foundation for further investigations into the mechanisms underlying the effects of AS-IV and CAG in diverse disease models and lay the groundwork for future clinical applications.

## 4 Application of AS-IV and CAG in regulating macrophage polarization for disease treatment

### 4.1 AS-IV: mediating macrophage phenotype switching

AS-IV, a natural plant-derived compound, has demonstrated significant therapeutic potential in recent years, particularly in the treatment of inflammatory, autoimmune, ischemic, and vascular diseases. Its primary mechanism of action involves modulating macrophage polarization and suppressing pro-inflammatory signaling pathways, thereby exerting anti-inflammatory, immunomodulatory, and tissue-protective effects.

#### 4.1.1 Inflammatory diseases

Inflammatory diseases, characterized by local or systemic inflammatory responses, often arise from abnormal macrophage activation and polarization. AS-IV has shown remarkable efficacy in various inflammatory disease models, particularly in regulating macrophage phenotypic transformation. Sepsis, a heterogeneous and life-threatening condition, remains one of the leading causes of mortality worldwide (Levy et al., 2003). The intestine is considered a critical “trigger” for sepsis, as sepsis-induced intestinal inflammation compromises the epithelial barrier, allowing harmful substances to infiltrate (Haussner et al., 2019). In a cecal ligation/puncture-induced sepsis model (n = 8), AS-IV (3 mg/kg) reversed M1 macrophage polarization and promoted M2 phenotypes, as shown by Western blot and quantitative real-time polymerase chain reaction (qRT-PCR) analyses. As summarized in Table 1, AS-IV suppresses M1 polarization in sepsis models via the NF-κB pathway. This was achieved by restoring intestinal microbiota balance, increasing short-chain fatty acid production (e.g., butyric acid), and inhibiting NLRP3 inflammasome activation, thereby reducing intestinal inflammation and epithelial barrier damage (Yang et al., 2024). Further studies by Liu et al. using a cecal ligation and puncture mouse model (n = 4) to determine Western blots and cytokines and chemokines and demonstrated that AS-IV inhibited LPS-induced macrophage overactivation by suppressing the NF-κB and extracellular signal-regulated kinase 1/2 signaling pathways, highlighting its potential for sepsis treatment (Liu et al., 2016). In a sepsis-associated acute liver injury model (n = 3), Western blot and RT-qPCR analyses showed that AS-IV (50 μg/g) modulated macrophage polarization and pyroptosis through activation of the AMPK/SIRT1 signaling pathway. This regulation reduced pro-inflammatory cytokines such as IL-6 and TNF-α, increased anti-inflammatory cytokines such as IL-10, and decreased pyroptosis-associated inflammatory factors, including IL-1β and gasdermin D, effectively mitigating liver inflammation and tissue damage (Kuang et al., 2024).

**TABLE 1:** The pharmacological effects of AS-IV and CAG on regulating macrophages.

Compound	Pharmacological activity	Disease	Animals/cells	Concentration/dose and duration	Effects on macrophages	Molecular target	References
AS-IV	Anti-inflammatory	Intestinal mucositis	Ara-C-induced mice LPS-induced RAW 264.7 cells	10–40 mg/kg/day for 10 days 50–200 μM for 24 h	↓M1	Akt signaling	Li et al. (2023a)
		Inflammatory bowel disease	DSS-induced mice IFN-γ^+^LPS/IL-4-stimulated BMDMs	50–100 mg/kg/day for 10 days 100 µM for 24 h	↓M1 ↑M2	STAT signaling	Tian et al. (2021)
		Sepsis	CLP-stimulated mice intestinal injury	3 mg/kg	↓Macrophage activation	NF-kB and ERK1/2 signaling pathways	Liu et al. (2016)
				100 mg/kg/day for 7 days	↓M1 ↑M2	NLRP3 inflammasome signaling pathway	Yang et al. (2024)
		Osteonecrosis of the femoral head	Glucocorticoid-induced mice IFN-γ/IL-4 -stimulated BMDMs	15 mg/kg/2 days for 28 days 200 nM for 48 h	↓M1 ↑M2	—	Jiang et al. (2021)
		Acute hepatic injury	LPS^+^D-Gal-induced mice LPS^+^IL-6-induced RAW 264.7 cells	50 μg/g 100 ng/mL for 4 h	↓M1 ↑M2 ↓Macrophage pyroptosis	AMPK/SIRT1 Signaling Pathway	Kuang et al. (2024)
		Rheumatoid arthritis	AIA rat model	100 mg/kg/day for 27 days	↓Macrophage activation	—	Wang and Chen (2014)
		Multiple sclerosis	EAE mice model	200 mg/(mL·kg)/day for 24 days	↓M1 ↑M2	TLR4/Myd88/NF-κB signaling pathway	Yu et al. (2023a)
		Abdominal aortic- aneurysm	Bap-/Ang II-induced mice	20–80 mg/kg/day for 42 days	↓Macrophage activation	Akt/NF-κB signaling pathway	Wang et al. (2018b)
		Ischemia-reperfusion injury	I/R-induced rat kidney injury IFN-γ^+^LPS/TGF-β1-induced RAW 264.7 cells	20 mg/kg/day for 7 days	↓M1 ↑M2	Hif-1α and NF-κB (p65)/Smad7 pathways	Tang et al. (2022a)
		Ischemic stroke	tMCAO rat model	40 mg/kg/day for 14 days	↓M1 ↑M2	PPARγ pathway	Li et al., (2021a)
		Diabetic wound	Streptozotocin-induced mice	1 µM for 5 days	↑M2	—	Luo et al. (2016)
		Atherosclerosis	WD-fed ApoE^−/−^ mice	10–50 mg/kg for 56 days	↓Macrophage adhesion and migration	TAK1 signaling pathway	Hua et al. (2025)
		Cigarette smoking-related lung injury	CSE-induced RAW264.7 cells	0–800 μg/mL for 24 h	↑Macrophage autophagy	TLR4/NF-κB signaling pathway	Ying et al. (2021)
		Spinal cord injury	SCI mice model LPS-induced HAPI cells	10 mg/kg/day until death 0–100 μM for 24 h	↓M1 ↑M2	mTORC1 signaling pathway	Lin et al. (2020)
		Myocardial infarction	MI mice model	40 mg/kg/day for 28 days	↓Macrophage pyroptosis	ROS/caspase-1/GSDMD signaling pathway	Zhang et al. (2022b)
		Liver fibrosis	CCl_4_-induced mice LPS-induced RAW 264.7 cells	20–100 mg/kg for 42 days 20–80 μM for 24 h	↓M1	Increasing FoxO1 expression	Hu et al. (2025)
	Anti-cancer	Lung cancer	LLC cell-bearing mice A549 and H1299 cells	40 mg/kg/day for 21 days 80 nM for 48 h	↓M2	AMPK signaling	Xu et al. (2018)
		Liver cancer	Huh-7 cell-bearing mice Huh-7 cells	20–100 mg/kg/ 3 days for 40 days 0–150 µM for 24 or 48 h	↓M2	TLR4/NF-κB/STAT3 signaling pathway	Min et al. (2022)
		Colorectal cancer	CT26 cell-bearing mice CT26 cells	15 mg/kg/3 days for 9 days 10–100 nM for 48 h	↓M2 ↑M1	—	Liu et al. (2020)
			MC38 cell-bearing mice MC38 cells	50 mg/kg/day for 21 days 0–200 µM for 48 h	↓M2 ↑M1	Inhibiting the release of Evs	Zhou et al. (2024)
		Ovarian cancer	SKOV3 cells	0.5–100 μg/mL for 24 h	↓M2	HMGB1-TLR4 axis	Wang et al. (2021c)
		Cervical cancer	HUVECs	0–80 µM for 24 h	↓M2	TGF-β/Smad2/3 signaling	Shen et al. (2023)
		Breast cancer	MDA-MB-231 cell-bearing mice MDA-MB-231 and MCF-7 cells	40 mg/kg/day for 20 days 5–100 µM for 24 h	↓M2	TGF-β-regulated Akt/ Foxo1 pathway	Yu et al. (2023b)
	Immunomodulation	Myelosuppression	CTX-induced mice RAW264.7 cells	25–100 mg/kg/day for 9 days 0–50 µM for 24 h	↑Macrophage immune activity	HIF-1α/NF-κB signaling pathway	Yao et al. (2023)
	Anti-aging	Senile osteoporosis	SOP mice RAW264.7 cells	10 mg/kg/day for 12 h 0–10 µM for 48 h	↓Macrophage senescence	STING/NF-κB pathway	Li et al. (2024c)
CAG	Anti-inflammatory	Psoriasis	IMQ-induced mice	12.5–50 mg/kg	↓Macrophage infiltration ↓Macrophage pyroptosis	NLRP3 inflammasome	Deng et al. (2019)
		Intracerebral hemorrhage	USA-induced mice LPS-induced BV2 cells	20–100 mg/kg/day for 3 days 10–40 µM	↓Macrophage migration	—	Chen et al. (2022b)

↓ indicates suppression; ↑ indicates activation.; AS-IV, Astragaloside IV; CAG, Cycloastragenol; Ara-C, Cytarabine; DSS, dextran sodium sulfate; AIA, adjuvant-induced arthritis; Bap, Benzopyrene; Ang-II, Angiotensin II; SCI, spinal cord injury; CSE, cigarette smoke extract; LLC, Lewis lung carcinoma; TLR4, Toll-like receptor 4; Myd88, myeloid differentiation factor 88; mTORC1, Mechanistic target of rapamycin complex 1; GSDMD, Gasdermin D; STAT, signal transducer and activator of transcription; NLRP3, NOD-, LRR- and pyrin domain-containing protein 3; PPARγ, peroxisome proliferator-activated receptor γ; AMPK, Adenosine 5′-monophosphate (AMP)-activated protein kinase; HMGB1, high mobility group box 1; STING, stimulator of interferon genes.

Beyond sepsis, AS-IV has shown efficacy in treating cytarabine-induced intestinal mucositis (Li J. J. et al., 2023). Western blot and immunoblotting analyses (n = 3) revealed that AS-IV suppressed excessive M1 macrophage activation by inhibiting the Akt signaling pathway, thereby reducing pro-inflammatory cytokines such as TNF-α and IL-6 and alleviating intestinal inflammation and tissue damage. In a mouse model, treatment with 40 mg/kg AS-IV over 10 days significantly reduced the number of CD86^+^ M1 macrophages in the intestine and minimized inflammatory infiltration. Similarly, in a dextran sulfate sodium-induced colitis model (n = 8), Tian et al. demonstrated that AS-IV at a dose of 100 mg/kg alleviated colon inflammation and tissue damage after 10 days of treatment. Molecular docking simulations and fluorescence analyses revealed that AS-IV promoted M1-to-M2 macrophage polarization by inhibiting the STAT1 signaling pathway and activating STAT3. This shift increased anti-inflammatory cytokine production and scavenger receptor expression while reducing pro-inflammatory cytokine secretion and enhancing macrophage phagocytosis, thereby facilitating tissue repair (Tian et al., 2021). Additionally, Jiang et al. reported that in an osteonecrosis of the femoral head mouse model, flow cytometry analysis showed that AS-IV (15 mg/kg) reduced the CD11b^+^F4/80^+^MHCII^+^ M1 macrophage phenotype and enhanced the CD11b^+^F4/80^+^CD206^+^ M2 macrophage phenotype. Enzyme-linked immunosorbent assay and Western blot analyses further indicated decreased TNF-α and IL-1β levels in the femoral head following AS-IV treatment. By attenuating the inflammatory response, AS-IV promoted osteocyte survival, facilitated tissue repair, and significantly improved arthritis symptoms (Jiang et al., 2021).

#### 4.1.2 Autoimmune diseases

AS-IV has shown considerable therapeutic potential in addressing autoimmune diseases such as rheumatoid arthritis and multiple sclerosis by modulating macrophage activation and polarization. In rheumatoid arthritis models, AS-IV exerts multidimensional anti-inflammatory effects. In a rat model of adjuvant-induced arthritis, Wang et al. demonstrated that administration of 100 mg/kg AS-IV over 27 days significantly suppressed the production of key inflammatory mediators, including IL-1β, TNF-α, and nitric oxide, which returned near baseline levels. This intervention alleviated adjuvant-induced arthritis-induced knee swelling and protected cartilage structure, effectively inhibiting arthritis progression while promoting tissue repair. Notably, colorimetric MTT assays confirmed that AS-IV was non-toxic to macrophages, further underscoring its safety profile (Wang and Chen, 2014).

In addition to its anti-inflammatory capabilities, AS-IV exhibits neuroprotective and immunoregulatory effects in multiple sclerosis, a progressive autoimmune disease marked by inflammatory demyelination in the CNS (GBD 2016 Neurology Collaborators, 2019). Using an experimental autoimmune encephalomyelitis mouse model, treatment with AS-IV (200 mg/mL·kg) delayed the onset of experimental autoimmune encephalomyelitis symptoms by approximately 2 days and resulted in a weight gain of 1.3 ± 0.27 g. Western blot analysis revealed significant suppression of pro-inflammatory cytokines IL-1β and TNF-α, coupled with elevated levels of the anti-inflammatory cytokine IL-10. Mechanistically, AS-IV inhibits the activation of M1 macrophages and microglia in the CNS by downregulating the TLR4/MyD88/NF-κB signaling pathway, facilitating their transition to the M2 anti-inflammatory phenotype. This shift enhances the release of neurotrophic factors and anti-inflammatory cytokines, which, in turn, promote oligodendrocyte progenitor cell differentiation and accelerate myelin repair. Through these mechanisms, AS-IV effectively mitigates disease progression, enhances neural repair, and improves overall neurological function (Yu J. et al., 2023).

#### 4.1.3 Ischemia and vascular diseases

AS-IV has demonstrated significant therapeutic potential in ischemic and vascular diseases, including abdominal aortic aneurysm (AAA), ischemia-reperfusion injury, and ischemic stroke. By modulating macrophage activation and polarization, AS-IV exerts anti-inflammatory, antioxidant, and tissue-protective effects. In the context of smoking-related AAA, AS-IV regulates macrophage activation by inhibiting the NF-κB signaling pathway. In a mouse model of AAA induced by 3,4-benzopyrene and angiotensin II, Wang et al. showed that treatment with 80 mg/kg AS-IV significantly reduced macrophage infiltration in the lesion area. This reduction was accompanied by decreased expression of pro-inflammatory cytokines, including CCL-1, IL-8, and TNF-α, suppression of NF-κB activity, and attenuation of oxidative stress and inflammatory responses, ultimately lowering the incidence of AAA to 33.3%. These effects are likely mediated through AS-IV’s regulation of Akt phosphorylation (Wang J. et al., 2018). Li et al. further explored the efficacy of AS-IV in cerebral ischemia and ischemic stroke. In a transient middle cerebral artery occlusion model (n = 4), 14 days of treatment with AS-IV at 40 mg/kg significantly improved neurological function, promoted brain tissue repair, and enhanced neurogenesis and angiogenesis. Western blot and qRT-PCR analyses revealed that AS-IV activated the PPARγ signaling pathway, which inhibited the expression of M1 macrophage and microglia markers (e.g., CD86, iNOS) and pro-inflammatory cytokines (TNF-α, IL-1β, IL-6). Concurrently, AS-IV increased the expression of M2 markers (e.g., CD206, arginase-1, YM1/2) and anti-inflammatory cytokines (IL-10, TGF-β), facilitating the transition of macrophages and microglia from an M1 pro-inflammatory phenotype to an M2 anti-inflammatory phenotype (Li L. et al., 2021).

Ischemia-reperfusion injury, a major cause of acute kidney injury, leads to early tissue damage and can progress to fibrosis, culminating in chronic kidney disease (Basile et al., 2016). Tang et al. demonstrated that AS-IV promoted the polarization of macrophages toward the M2 anti-inflammatory phenotype by modulating hypoxia-inducible factor-1α (Hif-1α), NF-κB (p65), and Smad7 signaling pathways, which are implicated in inflammation and fibrosis. In an ischemia-reperfusion-induced acute kidney injury model (n = 6), daily treatment with 20 mg/kg AS-IV for 7 days significantly reduced early pro-inflammatory cytokine release (I/R 24 h) and protected the kidney from acute injury. Additionally, Masson staining and immunohistochemical analysis of α-SMA revealed that AS-IV inhibited fibrosis-related signaling pathways, leading to reduced late-stage renal fibrosis (I/R 28 days) and limiting M2 macrophage infiltration (Tang L. et al., 2022). Collectively, these studies highlight AS-IV’s ability to comprehensively regulate macrophage phenotype transformation, cytokine secretion, and fibrosis-related signaling pathways, offering a promising therapeutic approach for ischemic and vascular diseases.

#### 4.1.4 Metabolic diseases

AS-IV has exhibited significant therapeutic potential in the management of metabolic diseases, including senile osteoporosis, diabetes, and atherosclerosis. Its primary mechanism of action involves modulating macrophage polarization to mitigate disease progression. The role of M2 macrophages is particularly critical in diabetic wound healing, where their anti-inflammatory properties facilitate tissue repair and regeneration (Goren et al., 2014). Luo et al. demonstrated that AS-IV enhanced neovascularization and accelerated wound healing in streptozotocin-induced diabetic mice. Treatment with 1 μM AS-IV for five consecutive days increased wound closure rates by 23.8% ± 1% after 10 days, as determined via two-color immunofluorescence analysis (n = 6). Additionally, the population of F4/80^+^CD206^+^ macrophages, indicative of M2 polarization, significantly increased, along with elevated levels of M2 macrophage activators such as IL-13 (Luo et al., 2016).

In senile osteoporosis, macrophage senescence and the aberrant secretion of inflammatory mediators such as calmodulin hinder the osteogenic differentiation of bone marrow mesenchymal stem cells while promoting adipogenic differentiation, thereby exacerbating bone loss (Li et al., 2022). Western blot analysis (n = 3) revealed that AS-IV suppressed the overactivation of the cyclic GMP-AMP synthase and stimulator of interferon genes signaling pathway in senescent macrophages, reducing the expression of inflammatory molecules, including TNF-α and iNOS. Concurrently, AS-IV upregulated M2 macrophage markers such as CD163 and CD206, restoring bone metabolism balance. Notably, 10 mg/kg AS-IV treatment for 12 h significantly downregulated genes associated with M1 macrophages in RAW264.7 cells and upregulated M2-associated genes. This transition inhibited macrophage senescence and enhanced the osteogenic differentiation capacity of bone marrow mesenchymal stem cells (Li M. et al., 2024).

Atherosclerosis, a prevalent metabolic disorder, is closely associated with abnormal macrophage activation. AS-IV mitigates this process by inhibiting the pro-inflammatory activation of endothelial cells via the TAK1 signaling pathway, thereby reducing macrophage adhesion and migration. Hua et al. investigated the effects of AS-IV in a mouse model of atherosclerosis (n = 6). Immunohistochemistry and immunofluorescence analyses demonstrated that 50 mg/kg AS-IV treatment for 56 days significantly reduced the size of atherosclerotic plaques. Furthermore, AS-IV suppressed the expression of pro-inflammatory factors such as intercellular cell adhesion molecule-1, vascular cell adhesion molecule 1, chemokine C-X-C ligand 1, and CCL5, while inhibiting macrophage-driven inflammatory activation and migration. This multifaceted regulation effectively curtailed the progression of atherosclerosis (Hua et al., 2025).

#### 4.1.5 Cancer

The potential of traditional Chinese medicine in targeting tumor angiogenesis and immunosuppressive tumor microenvironment has been highlighted in recent studies. For instance, Zhou et al. discussed the synergistic effects of co-targeting tumor angiogenesis and immunosuppressive tumor microenvironment using traditional Chinese medicine compounds, providing insights into the molecular mechanisms and therapeutic strategies for cancer treatment (Zhou et al., 2022). AS-IV has demonstrated potent anti-tumor effects by targeting the polarization of tumor-associated macrophages, a crucial component of the TME. Tumor-associated macrophages predominantly adopt an M2 phenotype that fosters angiogenesis, facilitates tumor cell invasion, and suppresses immune responses, thereby accelerating tumor progression (DeNardo and Ruffell, 2019). In lung cancer cell lines (A549 and H1299), Xu et al. showed through flow cytometry and quantitative PCR analyses that treatment with 40 mg/kg AS-IV significantly inhibited IL-13 and IL-4-induced M2 polarization. This was evidenced by reduced expression of CD206 and other M2-associated genes, as well as impaired invasion, migration, and angiogenesis mediated by M2 macrophages. Consistent with these findings, a lung cancer mouse model revealed that AS-IV inhibited tumor growth and metastasis. Western blot analyses indicated that AS-IV achieved these effects by modulating the AMPK signaling pathway, thereby reducing tumor-associated angiogenesis and invasion (Xu et al., 2018). In colorectal cancer models, AS-IV further demonstrated its anti-tumor efficacy by influencing TAM polarization. In the CT26 colon cancer cell model, AS-IV induced M2-to-M1 polarization, thereby suppressing tumor cell proliferation. Similarly, in the MC38 colorectal cancer model, AS-IV downregulated tumor-derived extracellular vesicles-mediated activation of M2 macrophages, leading to an increase in M1 macrophages and a reduction in colorectal cancer metastasis. After 98 h of treatment with 200 μM AS-IV, the TEV inhibition rate reached 76.46%, highlighting its potential to disrupt tumor-promoting macrophage functions (Liu et al., 2020; Zhou et al., 2024).

The anti-cancer effects of AS-IV have also been observed in HCC. In an HCC model, 100 mg/kg AS-IV significantly suppressed tumor cell migration, invasion, and proliferation by modulating the TLR4/NF-κB/STAT3 signaling pathway to inhibit M2 polarization (Min et al., 2022). Additionally, AS-IV has shown promise in female-specific cancers, including ovarian, breast, and cervical cancer. For ovarian cancer, AS-IV inhibited M2 polarization by inhibiting high mobility group protein B1 and TLR4 signaling, reduced migration and invasion of tumor cells (Wang X. et al., 2021). In breast cancer models, 40 mg/kg AS-IV attenuated M2 polarization by regulating the TGF-β/Akt-Foxo1 signaling pathway, effectively suppressing tumor growth (Yu Y. et al., 2023). For cervical cancer, AS-IV inhibited angiogenesis and epithelial-mesenchymal transition by inactivating the TGF-β/Smad2/3 signaling pathway, thereby preventing cancer cell proliferation and metastasis (Shen et al., 2023).

In summary, AS-IV can achieve anti-tumor effects at low doses. Across diverse cancer models, including lung cancer, colorectal cancer, and HCC, AS-IV effectively inhibits M2 macrophage polarization, downregulates M2-associated gene expression, and reduces tumor cell invasiveness, migration, and angiogenesis, thereby ameliorating pathological conditions. However, despite its promising therapeutic benefits, the mechanisms underlying AS-IV’s anti-cancer activity involve complex crosstalk among multiple signaling pathways and cytokines. Furthermore, *in vitro* experiments showed that AS-IV could reverse the M2 phenotype within 48 h, but *in vivo* continuous administration of AS-IV for 21 days was required to significantly inhibit tumor growth, suggesting that future dosage regimens need to be optimized. The precise molecular mechanisms governing its effects in different types warrant further elucidation.

#### 4.1.6 Other diseases

AS-IV has also demonstrated significant therapeutic potential across a diverse range of diseases. In a spinal cord injury model, treatment with 10 mg/kg AS-IV markedly attenuated inflammatory responses and reduced tissue damage. These effects were attributed to the inhibition of the mTORC1 signaling pathway, which promoted the polarization of microglia toward the M2 anti-inflammatory phenotype (Lin et al., 2020). Additionally, AS-IV has shown protective effects against smoking-induced lung injury. In RAW264.7 macrophages exposed to cigarette smoke extract, AS-IV modulated autophagy via the TLR4/NF-κB signaling pathway, resulting in diminished pro-inflammatory factor expression and enhanced autophagosome formation, effectively mitigating lung tissue damage (Ying et al., 2021). In a bone marrow suppression model, AS-IV improved hematopoietic function and enhanced macrophage immune activity by activating the HIF-1α/NF-κB signaling pathway. Enzyme-linked immunosorbent assay data revealed that administration of 100 mg/kg AS-IV over 9 days significantly enhanced the release of inflammatory mediators, such as nitric oxide, TNF-α, IL-6, and IL-1β, while simultaneously suppressing anti-inflammatory cytokines IL-10 and TGF-β1. This action facilitated the restoration of bone marrow immune function (Yao et al., 2023). Furthermore, AS-IV exhibited cardioprotective effects by reducing macrophage pyroptosis and minimizing myocardial cell damage (Zhang X. et al., 2022). Recent studies have also highlighted AS-IV’s role in mitigating peritoneal fibrosis induced by long-term peritoneal dialysis. AS-IV regulates miR-204-5p in macrophage-derived exosomes, targeting the Foxc1/β-catenin signaling pathway, thereby alleviating peritoneal fibrosis (Shan et al., 2024). Similarly, AS-IV has demonstrated efficacy in kidney diseases by reducing renal tubulointerstitial fibrosis (TIF) and mitigating kidney damage (Wang et al., 2024). In addition, AS-IV alleviates hepatic fibrosis by epigenetically regulating FoxO1 to inhibit macrophage glycolytic metabolism, thereby reducing M1 polarization and inflammatory responses (Hu et al., 2025). These effects were achieved by promoting the polarization of M1 macrophages toward the M2 phenotype and suppressing the production of pro-inflammatory factors.

**FIGURE 4:**
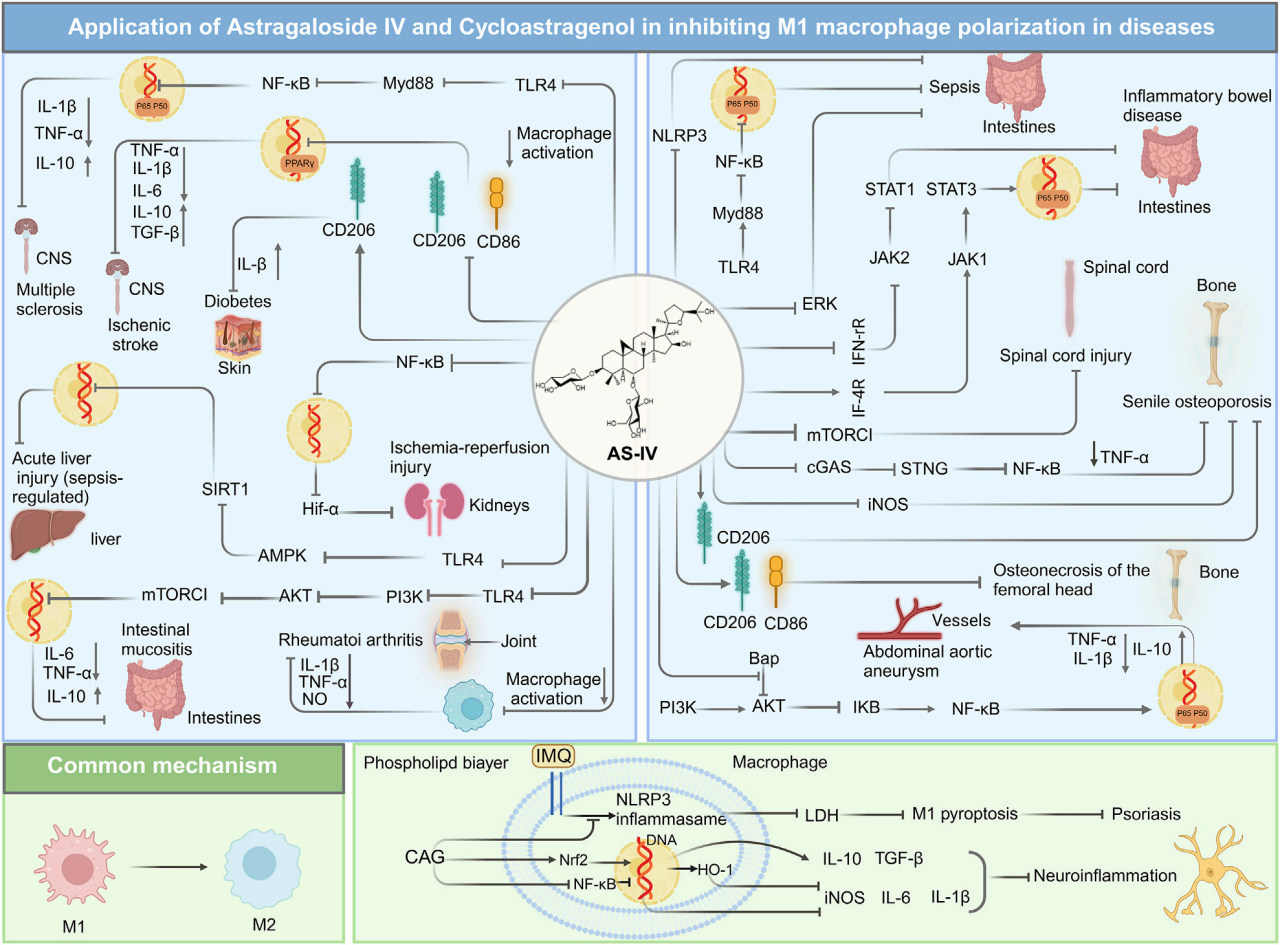
Overview of mechanisms and signaling pathways of astragaloside IV and cycloastragenol in alleviating diseases via regulation of M1 macrophage polarization.

**FIGURE 5:**
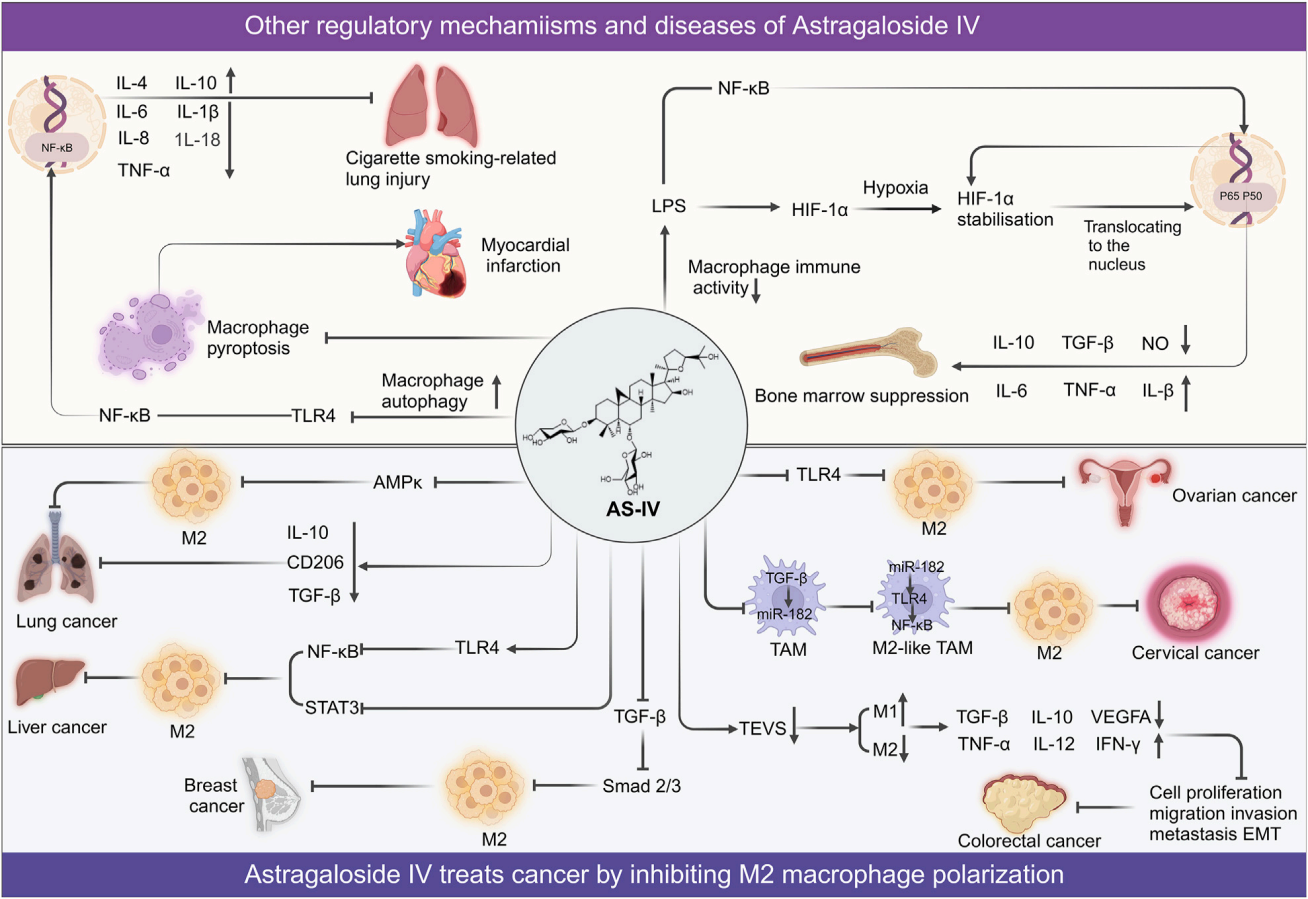
Overview of mechanisms and signaling pathways of astragaloside IV in alleviating diseases via regulation of M2 polarization and other macrophage function.

Collectively, these findings underscore the multifaceted role of AS-IV in modulating M1 (Figure 4) and M2 phenotypic transitions, as well as the functional regulation of macrophages (Figure 5) through intricate multi-pathway and multi-target mechanisms. These discoveries not only enrich our molecular understanding of AS-IV’s mechanisms of action but also pave the way for innovative macrophage-targeted therapeutic strategies.

### 4.2 CAG: regulating macrophage plasticity in inflammation and tissue

CAG, an important hydrolysate of AS-IV, has demonstrated significant therapeutic potential in various inflammation-related diseases. Its ability to modulate macrophage activation and polarization, attenuate inflammatory responses, and promote tissue repair underscores its pharmacological utility. These properties provide critical support for the clinical application of AS-IV and expand its therapeutic framework.

Psoriasis, a chronic inflammatory skin disorder with multifactorial genetic underpinnings, involves the dysregulation of both innate and adaptive immune pathways (Lowes et al., 2014). The recruitment and activation of macrophages within psoriatic lesions are pivotal in driving disease development and persistence. Notably, the infiltration of M1 macrophages exacerbates inflammation and tissue damage in affected skin (Clark and Kupper, 2006; Wang H. et al., 2009; Fuentes-Duculan et al., 2010). Western blot analyses revealed that CAG ameliorated imiquimod-induced psoriasis-like skin inflammation in mice by suppressing activation and pyroptosis of the NLRP3 inflammasome. At a dose of 50 mg/kg, CAG significantly improved the clinical score, reduced epidermal thickness, and mitigated histopathological changes in skin tissues. Additionally, it curtailed the production of pro-inflammatory cytokines, including IL-1β, TNF-α, and IL-6. CAG also decreased cortical thickening and inflammatory cell infiltration in psoriatic lesions. Further mechanistic studies indicated that CAG specifically reduced the infiltration of CD11b^+^ macrophages in the skin, while exerting minimal effects on dendritic cells, neutrophils, and T lymphocytes. By inhibiting the assembly of the NLRP3 inflammasome complex, CAG suppressed caspase-1 activation and gasdermin D-mediated pyroptosis, thereby mitigating the inflammatory cascade associated with psoriasis (Deng et al., 2019).

Beyond cutaneous inflammation, CAG has exhibited promise in treating neuroinflammation-related diseases by modulating macrophage polarization. It promotes the M2 anti-inflammatory phenotype in microglia and macrophages while inhibiting the pro-inflammatory M1 phenotype via activation of the nuclear factor erythroid 2-related factor 2 (Nrf2) signaling pathway and inhibition of NF-κB signaling. In LPS-treated BV-2 cells, Western blot analyses demonstrated that CAG significantly downregulated M1 macrophage markers while upregulating M2 markers. Immunofluorescence staining further revealed that CAG exerted these anti-inflammatory effects by suppressing NF-κB activation and enhancing Nrf2 expression alongside its downstream effector, heme oxygenase-1. Importantly, the regulatory effects of CAG on M2 markers were reversed upon Nrf2 knockdown with siRNA, underscoring the centrality of the Nrf2 pathway in its anti-inflammatory mechanism. Remarkably, CAG exhibits high bioavailability and permeability across the blood-brain barrier, enabling it to directly interact with specific molecular targets in microglia and macrophages, including protein Tyrosine Kinase 2, cell division cycle 42, and colony stimulating factor 1 receptor. These interactions inhibit the migration and proliferation of inflammatory cells, presenting therapeutic potential for neuroinflammatory conditions and secondary brain injury following intracerebral hemorrhage (Chen T. et al., 2022).

As a pivotal metabolite of AS-IV, CAG’ has garnered significant attention as a therapeutic candidate for inflammation-associated diseases, particularly due to its capacity to modulate M1 macrophage polarization, attenuate inflammatory responses, and promote tissue repair (Figure 4). The broad therapeutic potential of both AS-IV and CAG across diverse disease states underscores their critical role in regulating macrophage-driven pathology. Their applications in orchestrating macrophage polarization have revealed overlapping yet distinct mechanisms in various pathological models. *In vitro* studies consistently demonstrate their ability to suppress M1 polarization while enhancing markers characteristic of the M2 phenotype. Notably, AS-IV exhibits a broader anti-inflammatory effect in conditions such as sepsis and colitis, whereas CAG demonstrates pronounced efficacy in neuroinflammation and psoriasis, primarily through targeting the NLRP3 inflammasome. Despite these advances, important questions remain unanswered. The precise molecular pathways through which AS-IV regulates macrophage polarization have yet to be elucidated, as does the blood-brain barrier permeability of CAG and its long-term efficacy in chronic disease settings. Although *in vitro* findings strongly support their potential, they also reveal a dose-dependent dichotomy: AS-IV and CAG elicit therapeutic effects at μM concentrations over 48 h *in vitro*, yet *in vivo* efficacy requires mg-level dosing sustained over at least 3 days. This disparity underscores their metabolic processing within the body and highlights their low bioavailability. Table 1 encapsulates the pharmacological effects of AS-IV and CAG across diverse cell lines and murine models, providing a comprehensive overview of their therapeutic impact.

### 4.3 Synergistic effects of combination therapies involving AS-IV

Combination drug therapy has emerged as a powerful strategy to enhance the therapeutic efficacy of complex diseases by leveraging multi-target synergy. This approach not only lowers the required drug dosages but also minimizes toxic side effects and delays the onset of drug resistance (Hanahan et al., 2000; Blagosklonny, 2005). The multi-mechanistic nature of combination therapies offers unique advantages, particularly in the management of multifactorial diseases such as cancers and cardiovascular disorders.

AS-IV combined with tanshinone IIA shows synergistic anti-atherosclerotic effects. Both compounds mitigate the instability of atherosclerotic plaques by modulating macrophage-mediated inflammatory responses and the formation of foam cells. Their therapeutic efficacy is achieved by activating the PI3K/ Akt signaling pathway while inhibiting the TLR4/ NF-κB axis. In an ApoE^−/−^ mouse model of atherosclerosis, combined treatment with AS-IV (40 mg/kg) and tanshinone IIA (90 mg/kg) significantly reduced the expression of pro-inflammatory cytokines, including IL-6, TNF-α, and C-reactive protein, as well as MMP-9. Additionally, it increased the levels of the anti-inflammatory factor endothelial nitric oxide synthase. Further mechanistic studies revealed that blocking the PI3K/Akt pathway using the inhibitor Wortmannin led to increased nuclear translocation of NF-κB, elevated expression of TLR4, pro-inflammatory factors, and MMP-9, and suppressed endothelial nitric oxide synthase expression, thereby confirming that the PI3K/Akt signaling pathway plays a pivotal role in this combination treatment (Wang et al., 2020).

Similarly, the combination of AS-IV with anti-programmed cell death protein 1 immunotherapy has demonstrated enhanced anti-tumor activity in lung cancer. In a C57BL/6J mouse model of Lewis lung carcinoma, the combination of AS-IV (40 mg/kg) and anti-programmed cell death protein 1 antibody (10 mg/kg) significantly reduced tumor volume and weight. This combined therapy amplified the regulatory effects of AS-IV on macrophage polarization within the TME. Specifically, it upregulated the expression of the M1 macrophage marker mCD86 while downregulating the M2 macrophage marker mCD206, thereby reprogramming immunosuppressive M2 macrophages into tumor-suppressive M1 macrophages. This polarization shifts significantly enhanced the immune system’s capacity to target tumors. Furthermore, the combined therapy inhibited the activation of the PI3K/Akt and extracellular signal-regulated kinase pathways, thereby suppressing tumor cell proliferation and survival. It also promoted T cell activation, as evidenced by elevated expression of the T cell marker mCD3 and the activation marker mCD69 (Wu et al., 2024).

AS-IV and CAG have demonstrated broad therapeutic potential across diverse disease models, including inflammatory, metabolic, ischemic, vascular, autoimmune diseases, and cancers. By regulating macrophage polarization, these compounds effectively suppress inflammatory responses, promote tissue repair, ameliorate metabolic disorders, alleviate ischemic injury, and inhibit tumor growth and metastasis. The observed synergy between AS-IV and CAG in combination therapies paves the way for novel strategies in managing complex diseases. However, while AS-IV and CAG exhibit therapeutic effects across various preclinical disease models, their long-term efficacy and safety in humans remain to be fully established. Moreover, the dual role of macrophages in disease progression presents a challenge, as solely promoting or inhibiting the polarization of a single macrophage phenotype may not yield the desired therapeutic outcomes. Therefore, future studies should explore more nuanced approaches to macrophage modulation. Advancing research into the mechanisms and clinical applications of AS-IV and CAG will provide critical evidence to support their role in disease treatment and inform the development of innovative combination therapies.

## 5 Safety and pharmacokinetics

CAG and AS-IV are key bioactive components of *Astragalus* with promising therapeutic potential. Evaluating their safety and pharmacokinetic profiles is critical for assessing their clinical applicability. Current studies, primarily based on animal models and limited human trials, suggest favorable safety and metabolic characteristics; however, further research is required to support their widespread clinical use. Although clinical safety data for AS-IV and CAG remain limited, animal studies indicate that both compounds exhibit a high safety margin. In rat models, AS-IV demonstrated no significant toxicity within a dose range of 0.25–1.0 mg/kg. However, at a dose of 1.0 mg/kg/day, maternal toxicity was observed in pregnant rats, and fetal toxicity occurred at 0.5 mg/kg/day (Jiangbo et al., 2009). Xuying et al. reported that 1.0 mg/kg AS-IV inhibited maternal fertility and delayed developmental milestones in offspring, including fur development, eye opening, and cliff edge reflexes (Xuying et al., 2010). Consequently, caution is strongly advised when considering AS-IV use during pregnancy. Preliminary human trials have also confirmed the safety of AS-IV. No abnormalities in liver or kidney function, adverse reactions, or clinical toxicity were observed following a single intravenous injection of 600 mL or daily injections of 500 mL for seven consecutive days (Xu et al., 2013). Similarly, studies by Yu et al. demonstrated that *Astragalus* extract exhibited no detectable toxicity or adverse effects in rats and beagles (Yu et al., 2007). For CAG, toxicity studies further reinforced its safety profile. No treatment-related mortality or cardiac effects were observed in rats after oral administration of 150 mg/kg/day for 91 consecutive days. Additionally, CAG showed no genotoxicity or chromosomal aberrations in the mouse peripheral red blood cell micronucleus test or *in vitro* chromosome aberration assays, highlighting its robust safety characteristics (Szabo, 2014).

As a triterpenoid saponin, AS-IV exhibits low bioavailability. In rats, the oral bioavailability of 20 mg/kg AS-IV was only 3.66%, while in beagles, it was slightly higher at 7.4% (Gu et al., 2004; Zhang et al., 2007). Intravenous injection studies revealed that approximately 83% of AS-IV binds to plasma proteins in both rats and beagles, with a linear relationship observed within the concentration range of 250–1,000 ng/mL (Zhang et al., 2006). AS-IV is primarily absorbed through the intestine and distributed widely throughout the body, with the highest concentrations detected in the liver and kidneys (Chang et al., 2012). Its systemic clearance rate is relatively low (approximately 0.004 L/kg/min), indicating slow metabolism (Zhang et al., 2006). Interestingly, gender differences influence the elimination of half-life of AS-IV. In male rats, the elimination half-life ranged from 71.8 to 98.1 min, whereas in female rats, it varied from 34.0 to 131.6 min (Zhang et al., 2006). In contrast, CAG, a metabolite of AS-IV, demonstrates superior bioavailability and absorption efficiency. In rats, the oral bioavailability of CAG at 10 mg/kg was 25.70%, significantly surpassing that of AS-IV (2.2%) (Ma et al., 2017). CAG is efficiently absorbed through passive diffusion in the intestinal epithelium and undergoes extensive hepatic metabolism. Its systemic elimination rate (approximately 1,500 L/kg/min) is markedly higher than that of AS-IV, reflecting a faster metabolic rate (Ma et al., 2017). At doses of 10, 20, and 40 mg/kg, the elimination half-life of CAG in rats was 5.23 ± 1.55, 7.33 ± 3.03, and 6.06 ± 3.42 h, respectively, indicating relatively stable pharmacokinetic behavior (Ma et al., 2017). These pharmacokinetic differences underscore the enhanced metabolic efficiency of CAG compared to AS-IV. The pharmacokinetic parameters of AS-IV and CAG are summarized in Table 2.

**TABLE 2:** Pharmacokinetic parameters of AS-IV and CAG.

Compound	Animal	Mode of administration	Dosage (mg/kg)	Gender	Parameter						References
					T_max_ (h)	C_max_ (μg/mL)	t_1/2_ (h)	V_ss_ (L/kg)	CL (L/h·kg)	AUC_0∼∞_ (μg·h/mL)	
AS-IV	Rats	Oral	20	—	1.0 ± 0.5	0.0924 ± 0.0142	22.02 ± 8.26	—	0.045 ± 0.013	420.41 ± 129.01	Qing et al. (2019)
		Intravenous	0.75	Male	—	3.79	1.635	0.63	0.30	4.82	Zhang et al. (2006)
				Female	—	5.18	0.57	0.23	0.30	2.55	
			1.5	Male	—	6.98	1.12	0.71	0.42	6.70	
				Female	—	4.80	1.12	0.23	0.18	4.97	
			2	—	—	—	3.01	0.64	0.23	8.89	Du et al. (2005)
			2.5	—	—	—	3.37	—	0.42	5.99	Gu et al. (2004)
			3	Male	—	7.79	1.20	0.57	0.36	8.67	Zhang et al. (2006)
				Female	—	7.24	2.20	0.41	0.18	9.18	
		Oral	10	—	1.0	1.624	3.83	2.61	0.60	3.40	Zhang et al. (2007)
	Beagle dogs	Intravenous	0.25	Male	—	1.11 ± 0.28	0.87 ± 0.14	0.32 ± 0.13	0.24 ± 0.06	1.22 ± 0.52	Zhang et al. (2006)
				Female	—	1.13 ± 0.04	1.05 ± 0.37	0.36 ± 0.04	0.24 ± 0.06	1.10 ± 0.30	
			0.5	Male	—	4.39 ± 2.6	1.00 ± 0.14	0.32 ± 0.09	0.24 ± 0.06	2.60 ± 0.12	
				Female	—	3.48 ± 1.6	1.12 ± 0.13	0.31 ± 0.06	0.24 ± 0.06	2.88 ± 0.77	
			1	Male	—	7.92 ± 3.7	1.15 ± 0.35	0.28 ± 0.08	0.24 ± 0.06	6.03 ± 1.32	
				Female	—	8.86 ± 2.5	0.84 ± 0.22	0.23 ± 0.04	0.18 ± 0.06	5.92 ± 1.37	
			2	—	—	—	4.03	0.61	0.18	12.08	Zhang et al. (2007)
	Human	Intravenous	200 mL	—	1.08	2.12	2.14	—	—	4.38	Xu et al. (2013)
			300 mL	—	1.75	3.59 ± 0.46	2.59 ± 0.25	—	—	9.75 ± 1.31	
			400 mL	—	2.08	3.71 ± 0.32	2.62 ± 0.26	—	—	13.59 ± 1.90	Xu et al. (2013)
			500 mL	—	2.58	5.17 ± 1.27	2.69 ± 0.42	—	—	18.22 ± 5.60	
CAG	Rats	Oral	10	—	2.06 ± 0.58	180.95 ± 48.01	5.23 ± 1.55	—	7,780 ± 630	1.29 ± 1.03	Ma et al. (2017)
			20	—	1.48 ± 0.36	284.19 ± 139.17	7.33 ± 3.03	—	9,400 ± 5,130	2.98 ± 2.32	
			40	—	2.35 ± 1.17	566.67 ± 71.66	6.06 ± 3.42	—	9,230 ± 3,750	4.99 ± 2.22	
		Intravenous	10	—	—	—	1.68 ± 0.34	—	2,100 ± 5,600	5.02 ± 1.03	

AS-IV, Astragaloside IV; CAG, Cycloastragenol; Tmax, Peak time; Cmax, Peak concentration; CL, Clearance; t1/2, Clearance half-life; V, Volume of distribution; Vd, Apparent volume of distribution; Vss, Steady-state volume of distribution; AUC0∼∞, Area under the curve.

Collectively, these studies demonstrate that AS-IV and CAG exhibit favorable safety profiles and tolerability in animal models, with no significant toxicity observed in preliminary human trials. CAG, in particular, has shown high safety in toxicity and genotoxicity assessments. Pharmacokinetic studies reveal that CAG possesses higher bioavailability and metabolic efficiency than AS-IV, especially in terms of absorption and distribution, providing a strong foundation for its clinical development. However, the pharmacokinetics of AS-IV and CAG in humans remain incompletely understood. Further investigations are needed to elucidate their detailed metabolic pathways, potential drug interactions, and the effects of long-term use. Additionally, the maternal toxicity of AS-IV in pregnant mice and its potential impact on offspring warrant further exploration to clarify underlying mechanisms and establish safe usage guidelines.

## 6 Innovations in nanotechnology-driven delivery systems for AS-IV and CAG

**FIGURE 6:**
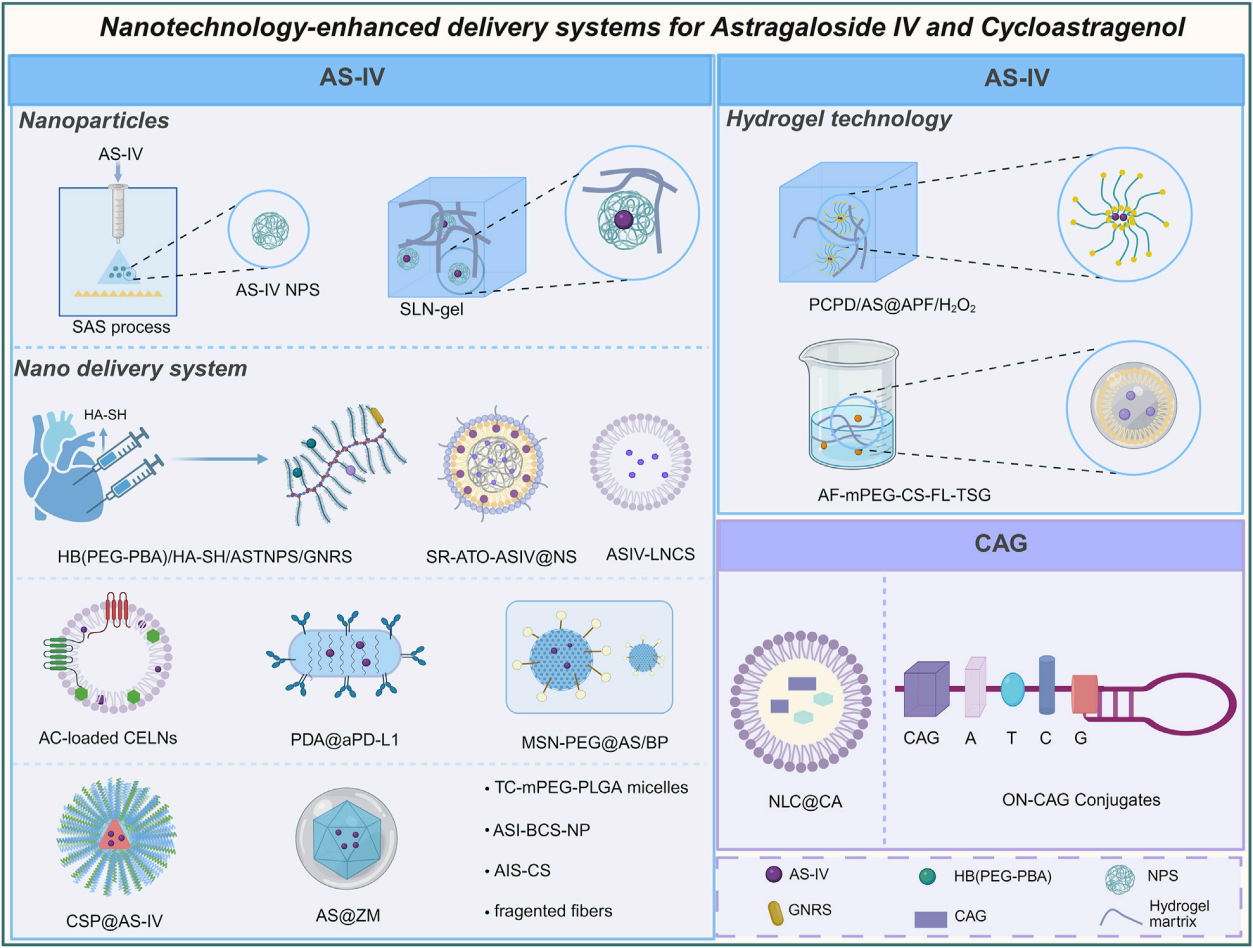
Astragaloside IV and cycloastragenol enhance bioavailability and other physicochemical properties by nanotechnology.

AS-IV and CAG, two natural products with diverse pharmacological activities, face significant clinical limitations due to their low water solubility and bioavailability. The advent of nanotechnology has provided a transformative approach to overcome these challenges by optimizing the pharmacokinetic properties of these compounds. Through the development of advanced nanocarriers and delivery strategies (Figure 6), researchers have markedly improved their solubility, absorption efficiency, and targeted delivery of AS-IV and CAG, thereby enhancing their therapeutic potential.

Nanoparticle-based technologies have demonstrated improved the bioavailability of AS-IV. For instance, nanoparticles of AS-IV prepared using supercritical anti-solvent technology reduced the particle size to 61.59 nm and altered the physical state from a crystalline structure to an amorphous form. This transformation increased the surface area in contact with water and significantly reduced crystallinity, resulting in a substantial improvement in solubility. Studies revealed that the cumulative dissolution of AS-IV increased from 16.8% to 41.4% within 24 h, with dissolution efficiency enhanced by 2.4-fold (Chen B. Q. et al., 2022). Another notable advancement involved the use of solid lipid nanoparticles combined with hydrogels (SLN-gel), which enabled a sustained-release effect. This system achieved a cumulative release rate of 70% over 48 h, effectively extending the drug’s duration of action *in vivo* (Chen et al., 2013). Recent studies utilizing copper silicate nanoparticles (CSPs) as carriers demonstrated pH-responsive degradation in the acidic joint microenvironment, achieving 90% cumulative drug release at pH 5.6 while maintaining structural stability under physiological conditions (Yang et al., 2025).

Nanotechnology has also significantly improved the targeted delivery of AS-IV, further enhancing its therapeutic efficacy. For example, AS-IV nanoparticles encapsulated with gold nanorods in a hydrogel matrix based on phenylboronic acid hyperbranched macromolecules enabled precise drug release at myocardial infarction sites via local injection. This approach minimized off-target drug distribution and releases only 15.2% of AS-IV at 72 h, thereby increasing drug concentration in target tissues. The as-prepared hydrogels also suggest a negligible cytotoxicity within 48 h (Chen J. R. et al., 2021). Recent studies on ZIF-8-based biomimetic nanoparticles (As@ZM) demonstrated significant bioavailability improvements for AS-IV. By encapsulating AS-IV within ZIF-8 nanoparticles and coating them with neutrophil membranes, the system achieved a 1.48-fold increase in bioavailability compared to free AS-IV, as evidenced by the AUC_0-t_ values (18.02 ± 0.88 vs. 12.19 ± 1.01 h μg/mL). This enhancement was attributed to neutrophil membrane-mediated targeting, which improved cellular uptake and reduced off-target clearance (Wu et al., 2025). Lipid nanocapsules have also been employed to enhance the oil solubility and biocompatibility of AS-IV. By incorporating phospholipids and surface polyethylene glycol modifications, lipid nanocapsules prolonged ocular retention time, reduced local clearance rates, and significantly improved bioavailability (Sun et al., 2020). Additionally, biomimetic nanosuspensions (SR-ATOASIV@NS) coated with SP94-modified red blood cell membranes and combined with arsenic trioxide and AS-IV demonstrated excellent sustained-release characteristics. At physiological pH (7.4), this system achieved a slow release of 52.74% over 48 h, effectively extending the drug’s circulation time and enhancing therapeutic efficacy (Raza et al., 2024). The development of novel nanocarriers has significantly advanced the delivery efficiency and therapeutic potential of AS-IV. For instance, tetracycline-grafted methoxy polyethylene glycol-polylactic acid-glycolic acid copolymer micelles (TC-mPEG-PLGA) loaded with AS-IV had a relative bioavailability of 218.9% and high affinity, which reduced the toxicity of AS-IV to non-target tissues (Que et al., 2022). Similarly, the use of polycaprolactone-methoxy polyethylene glycol and polycaprolactone-polyethylene glycol-aldehyde to synthesize AS-IV-loaded nanoparticles significantly enhanced bioavailability through sustained release (Kong et al., 2024). In addition, a two-dimensional black phosphorus nano-spray (MSN-PEG@AS/BP) was engineered by combining AS-IV with mesoporous silica nanoparticles. Compared with nano-silica, MSN effectively solves the problems of drug loading efficiency and sustained release kinetics due to its high specific surface area and ordered mesoporous channels. The system leverages MSN to achieve efficient drug loading and sustained release of AS-IV, offering a promising strategy for precise drug delivery (Liu et al., 2022; Zhang et al., 2017). Likewise, β-asarone modified AS-IV loaded chitosan nanoparticles (ASI-βCS-NP) achieved brain-targeted delivery through nasal administration, providing an efficient approach for treating neurological disorders (Zhao et al., 2023b). In recent *in vitro* cellular experiments, chitosan-based nanoparticles (ATS-CS) have also demonstrated exceptional drug-loading capabilities for AS-IV, achieving an encapsulation efficiency of 68.7% and a loading capacity of 13%. Notably, at a concentration of 17.5 μg/mL, ATS-CS exhibited equivalent suppression of TNF-α and IL-6 levels compared to 35 μg/mL of free AS-IV, indicating a 50% reduction in the effective dose due to enhanced cellular uptake efficiency (Qu et al., 2025). The turmeric nodule exosome-like nanoparticles (AC-CELN) loaded with astragalus components significantly increased the area under the plasma concentration-time curve of AS-IV by nearly 1.17-fold, thereby enhancing its absorption and bioavailability *in vivo* and ultimately improving its pharmacological effects (Yang et al., 2023). Another innovative carrier, fragmented fiber-based systems, demonstrated the capacity for sustained AS-IV release, with cumulative release rates approaching 80% over 25 days, significantly extending the drug’s duration of action (Li et al., 2015).

The integration of nanotechnology with gel-based systems has further optimized the application of AS-IV. For example, an injectable antibacterial and antioxidant hydrogel composed of poly (citric acid-co-polyethylene glycol) and dopamine enabled sustained AS-IV release over 300 h, achieving a total release of nearly 80% while exhibiting excellent antibacterial properties and biocompatibility (Zhao et al., 2023a). Additionally, thermosensitive sol-gel (TSG) systems extended the ocular retention time of AS-IV through electrostatic interactions between cations and anions on the corneal surface. These systems transition from liquid to semi-solid at body temperature, preventing sudden drug release, improving permeability, and enhancing relative bioavailability by 2.47-fold, thereby offering an innovative solution for the treatment of ocular diseases (Peng et al., 2023). Nanotechnology has also contributed to improving the bioavailability of CAG. Encapsulation of CAG in nanostructured lipid carriers enhanced its physical stability and bioavailability through rapid release kinetics, achieving a drug loading capacity of 26.7% (Chen et al., 2024). Furthermore, CAG was incorporated into oligonucleotide structures using a DNA synthesizer to form oligonucleotide-CAG conjugates. This approach not only improved the water solubility of CAG but also optimized its delivery efficiency and metabolic profile *in vivo*, providing critical support for its application in treating complex diseases (Tang L. M. et al., 2022). The specific applications of various nanotechnologies in the delivery of AS-IV and CAG are summarized in Table 3.

**TABLE 3:** Application of nanotechnology in AS-IV and CAG.

Nanotechnology	Compound	Route of administration	Drug loading	Findings	References
Bioactive Hydrogels	AS-IV	Local injection	—	The sustained release of AS-IV reached nearly 80% within 300 h.	Zhao et al. (2023a)
Fragmented nanofibers		Local injection	1.4%	The release of AS-IV reached 80% within 600 h.	Li et al., (2015)
ASIV-LNCs		Ocular instillation	90 nm, 506.4 μg/mL	The release of AS-IV reached 80% within 48 h.	Sun et al. (2020)
mPEG-CS-FL-TSG		Ocular administration	2.41%	The release of AS-IV was 57.09% within 4 h; The AUC of AS-IV was 2.47 times that of the AT-solution group (*p* < 0.01).	Peng et al. (2023)
SR-ATO-ASIV@NS		Intravenous injection	21.19%	The release of AS-IV was 52.74% ± 0.7% within 48 h.	Raza et al. (2024)
AC-CELNs		Gastric gavage (i.g.)	AC: CELNs (mg/mg) = 20:5 4,543.46 ± 95.14 (ng/mg)	The release of AS-IV was 87.62% ± 5.41% within 48 h, while the release of free drug reached 100.22% ± 7.60% within 6 h. The residues of formononetin in SGF and SIF were only 66% and 40%, respectively. The content of formononetin in CELNs remained almost unchanged within 2 h. The relative bioavailability (RBA) was 218.9%.	Yang et al. (2023)
TC-mPEG-PLGA		Tail vein injection	8.72% ± 0.09%	The release of AS-IV was 77.25% within 4 h, while the release of free AS-IV was 79.05%. The AUC of AS-IV was 28.260 h μg/mL, while that of free AS-IV was 12.912 h μg/mL.	Que et al. (2022)
MSN-PEG@AS/BP		Local spraying	8.12%	The release of AS-IV reached 91.32% within 96 h.	Liu et al. (2022)
ASI-βCS-NP		Intranasal administration (i.n.)	0.0140% ± 0.019%	The release of AS-IV was 20% within 48 h.	Zhao et al. (2023b)
SAS		Intravenous injection	—	The cumulative dissolution of AS-IV increased from 16.8% ± 4.5%–41.4% ± 2.8% within 24 h.	Chen et al. (2022a)
SLN-gel		Topical skin administration	9%	The release of AS-IV was 70% within 48 h.	Chen et al. (2013)
HB(PEG-PBA)/HA-SH/AST NPs/GNRs hydrogels		Intramyocardial injection	—	The release of AS-IV was 15.2% within 72 h.	Chen et al. (2021a)
CSP@AS-IV		Intra-articular injection	74.8%	The release of AS-IV was 90% within 48 h (pH = 5.6). The release of AS-IV was 30% within 12 h (pH = 7.4).	Yang et al. (2025)
As@ZM		Intravenous injection	—	The AUC_0-t_ of AS-IV was 18.02 ± 0.88 h μg/mL, while that of free AS-IV was 12.19 ± 1.01 h μg/mL. The bioavailability increased 1.48-fold.	Wu et al. (2025)
ATS-CS		—	13%	—	Qu et al. (2025)
PPA@aPD-L1		Tail vein injection	12.48%	—	Kong et al. (2024)
NLC@CAG	CAG	Oral gavage, intravenous injection	26.7%	—	Chen et al. (2024)
ON-CAG conjugate		—	—	—	Tang et al. (2022b)

AS-IV, Astragaloside IV; CAG, Cycloastragenol; AUC, Area under the curve.

In summary, nanotechnology has demonstrated immense potential in the drug development of AS-IV and CAG. By significantly enhancing solubility, bioavailability, and targeted delivery, these strategies have optimized the pharmacokinetic profiles of these natural products and expanded their clinical applications. Particularly in the treatment of inflammation, cardiovascular diseases, and neurological disorders, the combination of nanoparticles, gels, and sustained-release systems offers innovative solutions for the clinical translation of these compounds. However, challenges remain. The long-term stability and biocompatibility of nanocarriers require further refinement to ensure their safety and efficacy *in vivo*. Additionally, the complex preparation processes and high production costs of nanocarriers pose significant barriers to large-scale applications. Future research should focus on the development of intelligent, responsive nanocarriers, such as pH-sensitive or temperature-sensitive systems, which enable precise drug release and efficient targeted delivery tailored to pathological microenvironments. The use of biodegradable materials in nanocarrier design could further reduce immune response risks and improve the metabolic rate of drugs *in vivo*, thereby enhancing therapeutic outcomes. Moreover, the integration of multidisciplinary technologies, such as combining gene editing with nanotechnology, holds great promise for optimizing the delivery systems of AS-IV and CAG and improving their therapeutic efficacy in specific diseases. These innovative directions will pave the way for the clinical development of natural product-based drugs, offering precise and efficient solutions for the treatment of complex diseases.

## 7 Conclusion and outlook

### 7.1 Therapeutic potential and mechanisms of action of AS-IV and CAG

AS-IV and its hydrolysate, CAG, have demonstrated broad therapeutic potential across a range of diseases by modulating macrophage function. These natural compounds exhibit significant pharmacological effects, including anti-inflammatory, anti-tumor, anti-aging, and antioxidant properties, as evidenced by *in vitro* studies and mouse disease models. Notably, AS-IV and CAG play a pivotal role in regulating macrophage behavior, influencing their fate and functionality. By restoring macrophage polarization balance, inhibiting dysfunctional activation and aberrant migration, reducing inflammatory infiltration, and promoting autophagy, these compounds effectively improve the inflammatory microenvironment, repair tissue damage, and regulate TME. These effects are primarily mediated through the modulation of key signaling pathways, including TLR4/NF-κB, PI3K-STAT, PPARγ, AMPK, and mTORC1. The therapeutic potential of AS-IV and CAG has been validated in various preclinical models, encompassing inflammatory diseases, autoimmune disorders, ischemic injuries, metabolic conditions, neuroinflammation, and cancers. Interestingly, the combination of AS-IV with tanshinone IIA exhibits a marked synergistic effect in conditions such as atherosclerosis, enhancing the stabilization of vulnerable plaques while significantly amplifying antioxidant and anti-inflammatory activity. In recent years, advancements in nanotechnology have addressed the challenges posed by the low solubility and bioavailability of AS-IV and CAG, offering novel avenues to enhance their clinical applicability and therapeutic efficacy.

### 7.2 Challenges in clinical translation of AS-IV and CAG

At present, no marketed drugs containing AS-IV are available. Investigational products currently in development include AS-IV glucose injection and AS-IV sodium chloride injection (Acceptance numbers: X0408491 and X0408490), both targeting angina pectoris in coronary heart disease. However, their development remains at the completed Phase III clinical trial stage, with no subsequent updates reported (Chinese Clinical Trial Registry, 2015). In contrast, CAG has a commercially available formulation: Cycloastragenol 10 mg hard capsules, manufactured by Auzzie Lab. With a recommended daily dose of 10 mg, this formulation demonstrates potential to support cellular function and promote overall health and wellbeing (Australian Government Department of Health and Aged Care, 2018). Nevertheless, it is exclusively a single-compound drug. Comparatively, on 1 November 2019, ProBLEN introduced *Telomere-DNA*, a novel spray formulation. This product transiently enhances the body’s capacity to maintain or rebuild telomere length, support cellular function, and protect against DNA damage (U.S. Food and Drug Administration, n.d.). However, it remains an unapproved homeopathic product under current regulatory standards. Nevertheless, there are still certain challenges in clinical development. One of the primary obstacles lies in the incomplete understanding of their molecular mechanisms, particularly in regulating macrophage polarization. Macrophage polarization is a complex and dynamic process, involving the interplay of multiple signaling pathways and effector genes. Current research predominantly focuses on classical pathways, yet a comprehensive understanding of the binding interactions between specific ligands and receptors, as well as the multilayered regulation of downstream signal transduction, is still lacking. Modern gene-editing technologies, such as CRISPR-Cas9, offer powerful tools to elucidate these mechanisms. For instance, by constructing a single-guide RNA (sgRNA) library targeting 2,398 genes and applying genome-wide CRISPR-Cas9 screening in B16 tumor cells, researchers identified protein tyrosine phosphatase non-receptor type 2 as a novel cancer immunotherapy target. This study also highlighted the potential of CRISPR-Cas9 screening in identifying critical immunomodulatory genes (Manguso et al., 2017). In the future, similar approaches could be employed to systematically investigate how AS-IV influence macrophage transcriptional networks and epigenetic modifications through specific signaling pathways.

Another critical challenge is the incomplete characterization of the pharmacokinetic profiles of AS-IV, which are essential for optimizing dosing regimens and enhancing the therapeutic efficacy. Existing studies predominantly rely on animal models, and there is a pressing need to elucidate the absorption, distribution, metabolism, and excretion (ADME) processes across different administration routes, as well as the *in vivo* dynamics of their active metabolites. CADD presents a promising avenue for addressing these limitations. By leveraging the molecular structures of AS-IV, CADD can facilitate the development of novel compounds with improved bioavailability and targeted delivery. Recent studies, for example, synthesized two peptide compounds, AKVUAM-1 and AKVUAM-2, using CADD technology. These compounds exhibited water solubilities of 2.30 × 10^−7^ mol/L and 3.03 × 10^−8^ mol/L, respectively, significantly improving pharmacokinetic properties and successfully inhibiting interactions between *Mycobacterium tuberculosis* and macrophage surface receptors (Simsek et al., 2024). This research provides a theoretical and experimental foundation for designing high-efficiency, low-toxicity therapeutics, and similar strategies could be applied to optimize the molecular design of AS-IV. Despite promising results in animal studies, the long-term safety and efficacy of AS-IV in humans remain to be systematically validated through clinical trials. Notably, discrepancies in therapeutic doses reported in the literature underscore the need to establish a safe and effective dosing range before clinical application.

Furthermore, the development of AS-IV and CAG formulations faces several technical challenges, including improving stability, loading efficiency, and targeted delivery. Emerging nanotechnologies have shown great promise in overcoming these limitations. Drug delivery platforms incorporating nanostructured liposomes, polymer micelles, and thermosensitive gels have successfully enhanced the water solubility, delivery efficiency, and tissue specificity of AS-IV and CAG, thereby improved their metabolic kinetics and bolstered their clinical competitiveness.

### 7.3 Future directions for AS-IV and CAG research

Future research and development of AS-IV and CAG can be advanced along three primary directions. Initially, existing studies in cancer treatment and immune regulation suggest that combination therapies can significantly amplify therapeutic outcomes. Thus, exploring synergistic strategies that combine AS-IV or CAG with immune checkpoint inhibitors, tumor vaccines, and other immunotherapies represents a promising avenue for cancer treatment (Liu X. et al., 2021). For instance, AS-IV has been formulated into excipient-free mesoporous microfibers (SMF) and utilized as an injectable three-dimensional (3D) scaffold for tumor vaccine delivery. This approach demonstrated a remarkable enhancement in immune efficacy, reducing tumor weight by an average of 58.47%. Moreover, SMF has shown the potential to synergize with clinically established TLR-4 agonists (e.g., MPLA) and immune checkpoint blockers such as anti-PD-L1, thereby further augmenting anti-tumor effects (Huo et al., 2024). Additionally, photodynamic therapy, which eliminates tumor cells and pathogens by generating reactive oxygen species (ROS), has shown efficacy in treating chromoblastomycosis (CBM) (Yi et al., 2017). Given its role in modulating macrophage phagocytosis and autophagy, combining AS-IV with photodynamic therapy may further enhance therapeutic outcomes by boosting macrophage immune function.

Subsequently, macrophages serve as the primary host cells for *M. tuberculosis*, and regulating macrophage polarization to promote M1 polarization or reverse M2 polarization has emerged as a key strategy in tuberculosis treatment (Castaño et al., 2011). AS-IV exhibits unique advantages in modulating macrophage polarization and enhancing anti-infective immunity, making it a promising candidate for developing novel anti-tuberculosis immunotherapies. For example, gene knockout studies have identified the ROS-ATM-Chk2 signaling axis as a critical mediator of M1 macrophage polarization, with inhibition of this axis significantly reducing lung inflammation (Li C. et al., 2024). By leveraging AS-IV’s ability to balance M1/M2 macrophage functions, its therapeutic potential in tuberculosis could be further explored. Furthermore, AS-IV and CAG may strengthen host defenses against other infectious pathogens by modulating macrophage immunometabolism pathways, providing a new direction for infectious disease research (Yao et al., 2023).

Lastly, AS-IV and CAG hold promises for treating complex diseases such as metabolic disorders and neuroinflammation. In metabolic diseases, these compounds exhibit anti-inflammatory and metabolic regulatory effects by modulating immune cell function and metabolic pathways. For instance, AS-IV improves insulin sensitivity by activating the AMPK signaling pathway and mitigates metabolic disturbances induced by high-fat diets (Wang C. Y. et al., 2018). In neuroinflammation, AS-IV and CAG have been shown to reduce neuroinflammatory responses by regulating microglial polarization, thereby alleviating the pathological progression of neurodegenerative diseases (Bao et al., 2023; Ikram et al., 2021). The integration of precision medicine technologies could further expand the clinical applicability of AS-IV and CAG, offering novel strategies for personalized treatment approaches. Concurrently, the development of multifunctional delivery platforms, such as nanotechnology-based targeted delivery systems, could enhance the stability and bioavailability of AS-IV and CAG, facilitating their translational applications in the treatment of complex diseases (Que et al., 2022).

**FIGURE 7:**
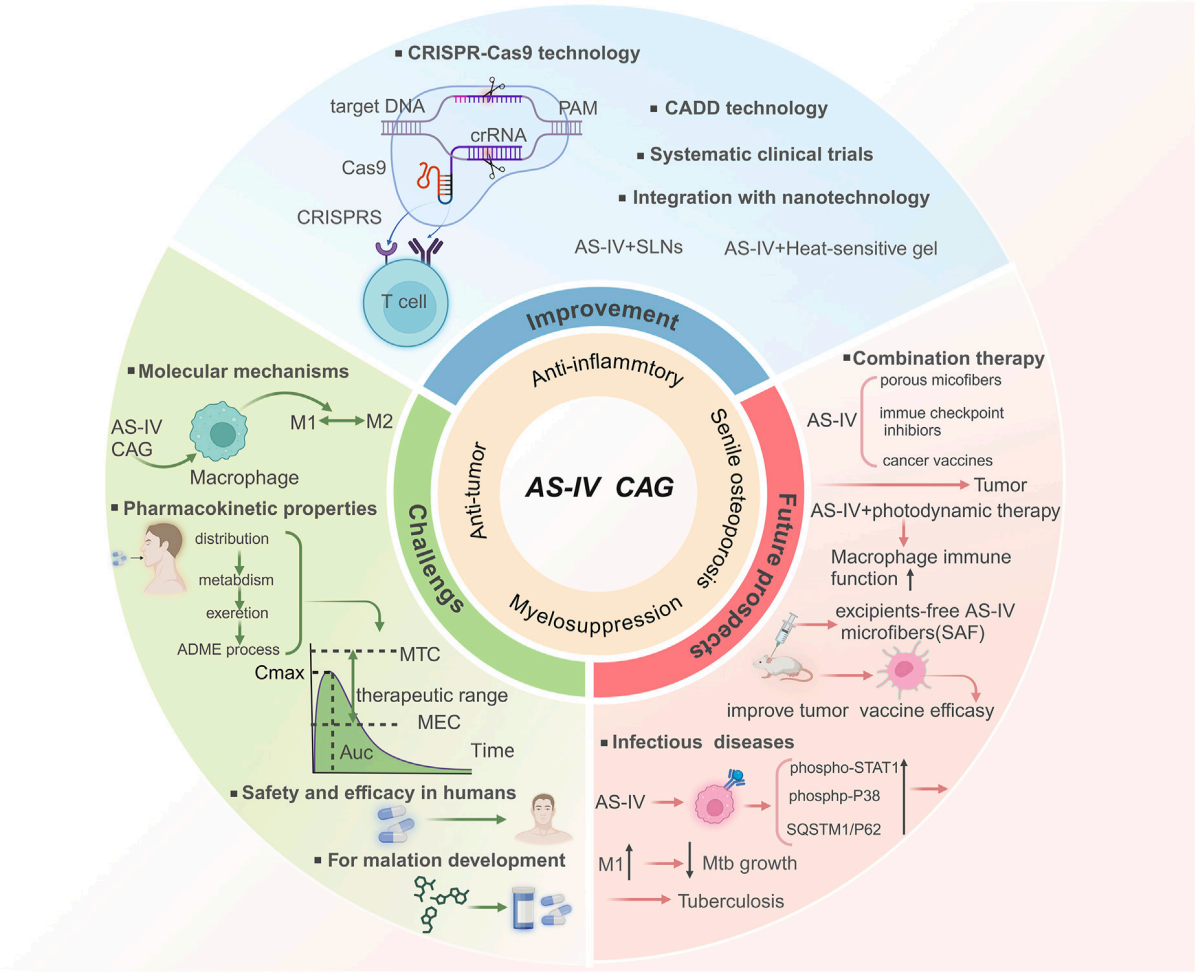
Summary of the research progress and future perspectives of astragaloside IV and cycloastragenol.

In summary, AS-IV and CAG, as bioactive compounds derived from natural plants, exhibit significant therapeutic potential across a wide range of pathological conditions, including inflammatory diseases, cancers, and infectious diseases. Through the application of modern biotechnologies such as gene editing, CADD, and high-throughput screening, coupled with advancements in nanotechnology, future research is expected to precisely define their molecular targets, optimize drug delivery efficiency, and validate their broad clinical applicability (Figure 7). These efforts will not only advance the field of natural medicine but also provide innovative strategies and tools for addressing complex diseases, thereby opening new frontiers in medical research.

## Abbreviations

AS-IV, astragaloside IV; CAG, cycloastragenol; Akt, protein kinase B; mTORC1, mammalian target of rapamycin complex 1; STAT, signal transducer and activator of transcription; NLRP3, NOD-like receptor family pyrin domain containing 3; TAK, TGF-β-activated kinase; TLR4, toll-like receptor 4; LPS, lipopolysaccharide; NF-κB, nuclear factor kappa B; TNF-α, tumor necrosis factor α; IL, interleukin; iNOS, inducible nitric oxide synthase; MyD88, myeloid differentiation primary response protein 88; IRAK, IL-1R-associated kinase; TRAF, tumor necrosis factor receptor-associated factor; TGF-β, transforming growth factor β; IFN-γ, interferon γ; JAK, janus kinase; PPAR, peroxisome proliferator-activated receptors; PI3K, phosphatidylinositol 3-kinase; AMPK, adenosine monophosphate-activated protein kinase; Th, T helper cell; RA, rheumatoid arthritis; CNS, central nervous system; TME, tumor microenvironment; CCL, C-C motif chemokine ligand; MMP, matrix metalloproteinase; qRT-PCR, quantitative real-time polymerase chain reaction; AAA, abdominal aortic aneurysm; HCC, hepatocellular carcinoma; Nrf2, nuclear factor erythroid 2-related factor 2; CADD, computer-aided drug design.

## References

[B1] Allavena P. Sica A. Solinas G. Porta C. Mantovani A. (2008). The inflammatory micro-environment in tumor progression: the role of tumor-associated macrophages. Crit. Rev. Oncol. Hematol. 66, 1–9. 10.1016/j.critrevonc.2007.07.004 17913510

[B2] Annamalai R. T. Turner P. A. Carson W. F. Levi B. Kunkel S. Stegemann J. P. (2018). Harnessing macrophage-mediated degradation of gelatin microspheres for spatiotemporal control of BMP2 release. Biomaterials 161, 216–227. 10.1016/j.biomaterials.2018.01.040 29421557 PMC5831261

[B3] Augustin J. M. Kuzina V. Andersen S. B. Bak S. (2011). Molecular activities, biosynthesis and evolution of triterpenoid saponins. Phytochemist. 72, 435–457. 10.1016/j.phytochem.2011.01.015 21333312

[B4] Austermann J. Roth J. Barczyk-Kahlert K. (2022). The good and the bad: monocytes’ and macrophages’ diverse functions in inflammation. Cells 11, 1979. 10.3390/cells11121979 35741108 PMC9222172

[B5] Australian Government Department of Health and Aged Care (2018). Cycloastragenol 10 mg (300387). Available online at: https://www.tga.gov.au/resources/artg/300387 (Accessed April 17, 2025).

[B6] Bao M. L. Bade R. Liu H. Tsambaa B. Shao G. Borjigidai A. (2023). Astragaloside IV against Alzheimer’s disease via microglia-mediated neuroinflammation using network pharmacology and experimental validation. Eur. J. Pharmacol. 957, 175992. 10.1016/j.ejphar.2023.175992 37598923

[B7] Basile D. P. Bonventre J. V. Mehta R. Nangaku M. Unwin R. Rosner M. H. (2016). Progression after AKI: understanding maladaptive repair processes to predict and identify therapeutic treatments. J. Am. Soc. Nephrol. 27, 687–697. 10.1681/ASN.2015030309 26519085 PMC4769207

[B8] Bernsmeier C. van der Merwe S. Périanin A. (2020). Innate immune cells in cirrhosis. J. Hepatol. 73, 186–201. 10.1016/j.jhep.2020.03.027 32240716

[B9] Blagosklonny M. V. (2005). Overcoming limitations of natural anticancer drugs by combining with artificial agents. Trends Pharmacol. Sci. 26, 77–81. 10.1016/j.tips.2004.12.002 15681024

[B10] Cao X. Li B. Chen J. Dang J. Chen S. Gunes E. G. (2021). Effect of cabazitaxel on macrophages improves CD47-targeted immunotherapy for triple-negative breast cancer. J. Immunother. Cancer 9, e002022. 10.1136/jitc-2020-002022 33753567 PMC7986678

[B11] Castaño D. Barrera L. F. Rojas M. (2011). *Mycobacterium tuberculosis* alters the differentiation of monocytes into macrophages *in vitro* . Cell. Immunol. 268, 60–67. 10.1016/j.cellimm.2011.02.006 21420074

[B12] Chang Y. X. Sun Y. G. Li J. Zhang Q. H. Guo X. R. Zhang B. L. (2012). The experimental study of Astragalus membranaceus on meridian tropsim: the distribution study of astragaloside IV in rat tissues. J. Chromatogr. B 911, 71–75. 10.1016/j.jchromb.2012.10.024 23217309

[B13] Chen B. Q. Liu H. Zhao Y. Lu X. C. Zhang C. Y. Kankala R. K. (2022a). Preparation of astragaloside IV (AS-IV) nanoparticles via SAS process for anticancer efficacy: optimization based on box-behnken design. J. Supercrit. Fluids 188, 105650. 10.1016/j.supflu.2022.105650

[B14] Chen D. Song T. T. Liu Y. Wang Y. Qin B. Y. Zhang Q. Y. (2024). Effective hydrogel vascular patch dual-loaded with cycloastragenol nanostructured lipid carriers and doxycycline for repairing extravascular injury in abdominal aortic aneurysm. Adv. Healthc. Mater 14, e2402497. 10.1002/adhm.202402497 39703126

[B15] Chen J. R. Han X. X. Deng J. Zhang J. Li L. Ni J. Y. (2021a). An injectable hydrogel based on phenylboronic acid hyperbranched macromer encapsulating gold nanorods and astragaloside IV nanodrug for myocardial infarction. Chem. Eng. J. 413, 127423. 10.1016/j.cej.2020.127423

[B16] Chen K. Zhang M. Gao B. Hasan A. Li J. Bao Y. (2023a). Characterization and protein engineering of glycosyltransferases for the biosynthesis of diverse hepatoprotective cycloartane-type saponins in Astragalus membranaceus. Plant Biotechnol. J. 21, 698–710. 10.1111/pbi.13983 36529909 PMC10037152

[B17] Chen K. Zhang M. Xu L. Yi Y. Wang L. Wang H. (2023b). Identification of oxidosqualene cyclases associated with saponin biosynthesis from Astragalus membranaceus reveals a conserved motif important for catalytic function. J. Adv. Res. 43, 247–257. 10.1016/j.jare.2022.03.014 36585112 PMC9811366

[B18] Chen Q. S. Bao L. L. Lv L. Xie F. Y. Zhou X. W. Zhang H. (2021b). Schisandrin B regulates macrophage polarization and alleviates liver fibrosis via activation of PPARγ. Ann. Transl. Med. 9, 1500. 10.21037/atm-21-4602 34805362 PMC8573433

[B19] Chen T. Li Z. Li S. Zou Y. Gao X. Shu S. (2022b). Cycloastragenol suppresses M1 and promotes M2 polarization in LPS-stimulated BV-2 cells and ischemic stroke mice. Int. Immunopharmacol. 113, 109290. 10.1016/j.intimp.2022.109290 36252498

[B20] Chen X. Peng L. H. Shan Y. H. Li N. Wei W. Yu L. (2013). Astragaloside IV-loaded nanoparticle-enriched hydrogel induces wound healing and anti-scar activity through topical delivery. Int. J. Pharm. 447, 171–181. 10.1016/j.ijpharm.2013.02.054 23500766

[B21] Chiang C. F. Chao T. T. Su Y. F. Hsu C. C. Chien C. Y. Chiu K. C. (2017). Metformin-treated cancer cells modulate macrophage polarization through AMPK-NF-κB signaling. Oncotarget 8, 20706–20718. 10.18632/oncotarget.14982 28157701 PMC5400538

[B22] Chinese Clinical Trial Registry (2015). Phase III clinical trial of Astragaloside IV glucose on stable angina of coronary artery disease. Available online at: https://www.chictr.org.cn/showproj.html?proj=7574 (Accessed April 15, 2025).

[B23] Ciesielska A. Matyjek M. Kwiatkowska K. (2021). TLR4 and CD14 trafficking and its influence on LPS-induced pro-inflammatory signaling. Cell. Mol. Life Sci. 78, 1233–1261. 10.1007/s00018-020-03656-y 33057840 PMC7904555

[B24] Clark R. A. Kupper T. S. (2006). Misbehaving macrophages in the pathogenesis of psoriasis. J. Clin. Invest. 116, 2084–2087. 10.1172/JCI29441 16886055 PMC1523394

[B25] Covarrubias A. J. Aksoylar H. I. Horng T. (2015). Control of macrophage metabolism and activation by mTOR and Akt signaling. Semin. Immunol. 27, 286–296. 10.1016/j.smim.2015.08.001 26360589 PMC4682888

[B26] Dai L. R. Tao Y. R. Shi Z. M. Liang W. L. Hu W. H. Xing Z. (2022). SOCS3 Acts as an Onco-immunological biomarker with value in assessing the tumor microenvironment, pathological staging, histological subtypes, therapeutic effect, and prognoses of several types of cancer. Front. Oncol. 12, 881801. 10.3389/fonc.2022.881801 35600392 PMC9122507

[B27] Deguine J. Barton G. M. (2014). MyD88: a central player in innate immune signaling. F1000prime Rep. 6, 97. 10.12703/P6-97 25580251 PMC4229726

[B28] DeNardo D. G. Ruffell B. (2019). Macrophages as regulators of tumour immunity and immunotherapy. Nat. Rev. Immunol. 19, 369–382. 10.1038/s41577-019-0127-6 30718830 PMC7339861

[B29] Deng G. Chen W. Wang P. Zhan T. Zheng W. Gu Z. (2019). Inhibition of NLRP3 inflammasome-mediated pyroptosis in macrophage by cycloastragenol contributes to amelioration of imiquimod-induced psoriasis-like skin inflammation in mice. Int. Immunopharmacol. 74, 105682. 10.1016/j.intimp.2019.105682 31203155

[B30] Ding Y. X. Ou-Yang X. Shang C. H. Ren A. Shi L. Li Y. X. (2008). Molecular cloning, characterization, and differential expression of a farnesyl-diphosphate synthase gene from the basidiomycetous fungus Ganoderma lucidum. Biosci. Biotechnol. Biochem. 72, 1571–1579. 10.1271/bbb.80067 18540102

[B31] Du L. Zhang Y. Chen Y. Zhu J. Yang Y. Zhang H. L. (2017). Role of microglia in neurological disorders and their potentials as a therapeutic target. Mol. Neurobiol. 54, 7567–7584. 10.1007/s12035-016-0245-0 27830532

[B32] Du S. Li C. Lu Y. Lei X. Zhang Y. Li S. (2019). Dioscin alleviates crystalline silica-induced pulmonary inflammation and fibrosis through promoting alveolar macrophage autophagy. Theranostics 9, 1878–1892. 10.7150/thno.29682 31037145 PMC6485284

[B33] Du Y. Zhang Q. Chen G. G. Wei P. Tu C. Y. (2005). Pharmacokinetics of astragaloside IV in rats by liquid chromatography coupled with tandem mass spectrometry. Eur. J. Drug Metab. Pharmacokinet. 30, 269–273. 10.1007/BF03190631 16435572

[B34] Duan Y. Du W. Song Z. Chen R. Xie K. Liu J. (2023). Functional characterization of a cycloartenol synthase and four glycosyltransferases in the biosynthesis of cycloastragenol-type astragalosides from Astragalus membranaceus. Acta Pharm. Sin. B 13, 271–283. 10.1016/j.apsb.2022.05.015 36815054 PMC9939298

[B35] Durham G. A. Williams J. J. Nasim M. T. Palmer T. M. (2019). Targeting SOCS proteins to control JAK-STAT signalling in disease. Trends Pharmacol. Sci. 40, 298–308. 10.1016/j.tips.2019.03.001 30948191

[B36] Dzik J. (2021). Metabolic evolutionary roots of the macrophage immune response in amoeba-bacteria interactions: the conserved role of hypoxia-induced factor and AMP kinase. Acta Biochim. Pol. 68, 457–476. 10.18388/abp.2020_5683 34374500

[B37] Feng L. Lo H. Zheng J. Weng W. Sun Y. Pan X. (2024). Cycloastragenol reduces microglial NLRP3 inflammasome activation in Parkinson's disease models by promoting autophagy and reducing Scrib-driven ROS. Phytomedicine 135, 156210. 10.1016/j.phymed.2024.156210 39522252

[B38] Feng L. M. Lin X. H. Huang F. X. Cao J. Qiao X. Guo D. A. (2014). Smith degradation, an efficient method for the preparation of cycloastragenol from astragaloside IV. Fitoterapia 95, 42–50. 10.1016/j.fitote.2014.02.014 24613799

[B39] Fu J. Wang Z. Huang L. Zheng S. Wang D. Chen S. (2014). Review of the botanical characteristics, phytochemistry, and pharmacology of Astragalus membranaceus (Huangqi). Phytother. Res. 28, 1275–1283. 10.1002/ptr.5188 25087616

[B40] Fuentes-Duculan J. Suárez-Fariñas M. Zaba L. C. Nograles K. E. Pierson K. C. Mitsui H. (2010). A subpopulation of CD163-positive macrophages is classically activated in psoriasis. J. Invest. Dermatol. 130, 2412–2422. 10.1038/jid.2010.165 20555352 PMC2939947

[B41] GBD 2016 Neurology Collaborators (2019). Global, regional, and national burden of neurological disorders, 1990-2016: a systematic analysis for the global burden of disease study 2016. Lancet Neurol. 18, 459–480. 10.1016/S1474-4422(18)30499-X 30879893 PMC6459001

[B42] Goren I. Pfeilschifter J. Frank S. (2014). Uptake of neutrophil-derived Ym1 protein distinguishes wound macrophages in the absence of interleukin-4 signaling in murine wound healing. Am. J. Pathol. 184, 3249–3261. 10.1016/j.ajpath.2014.08.011 25307347

[B43] Gu Y. Wang G. Pan G. Fawcett J. P. A J. Sun J. (2004). Transport and bioavailability studies of astragaloside IV, an active ingredient in radix astragali. Basic Clin. Pharmacol. Toxicol. 95, 295–298. 10.1111/j.1742-7843.2004.t01-1-pto950508.x 15569275

[B44] Guo J. N. Chen D. Deng S. H. Huang J. R. Song J. X. Li X. Y. (2022). Identification and quantification of immune infiltration landscape on therapy and prognosis in left- and right-sided colon cancer. Cancer Immunol. Immunother. 71, 1313–1330. 10.1007/s00262-021-03076-2 34657172 PMC9122887

[B45] Hanahan D. Bergers G. Bergsland E. (2000). Less is more, regularly: metronomic dosing of cytotoxic drugs can target tumor angiogenesis in mice. J. Clin. Invest. 105, 1045–1047. 10.1172/JCI9872 10772648 PMC300842

[B46] Hao S. Meng J. Zhang Y. Liu J. Nie X. Wu F. (2017). Macrophage phenotypic mechanomodulation of enhancing bone regeneration by superparamagnetic scaffold upon magnetization. Biomaterials 140, 16–25. 10.1016/j.biomaterials.2017.06.013 28623721

[B47] Haussner F. Chakraborty S. Halbgebauer R. Huber-Lang M. (2019). Challenge to the intestinal mucosa during sepsis. Front. Immunol. 10, 891. 10.3389/fimmu.2019.00891 31114571 PMC6502990

[B48] He F. Zhu Y. He M. Zhang Y. (2008). Molecular cloning and characterization of the gene encoding squalene epoxidase in Panax notoginseng. DNA Seq. 19, 270–273. 10.1080/10425170701575026 17852349

[B49] Hegazy M.-E. F. Mohamed T. A. ElShamy A. I. Mohamed A.-E.-H. H. Mahalel U. A. Reda E. H. (2015). Microbial biotransformation as a tool for drug development based on natural products from mevalonic acid pathway: a review. J. Adv. Res. 6, 17–33. 10.1016/j.jare.2014.11.009 25685541 PMC4293675

[B50] Hu Y. Kuang M. Song H. Tan Y. Zhou F. Pei G. (2025). Astragaloside IV prevents liver fibrosis by blocking glycolysis-mediated macrophage M1 polarization. Eur. J. Pharmacol. 995, 177353. 10.1016/j.ejphar.2025.177353 39971227

[B51] Hua S. Zhang H. Li J. Zhou X. Zhang S. Zhu Y. (2025). Astragaloside IV ameliorates atherosclerosis by targeting TAK1 to suppress endothelial cell proinflammatory activation. Int. Immunopharmacol. 146, 113842. 10.1016/j.intimp.2024.113842 39706043

[B52] Huang D. Gao W. Lu H. Qian J. Y. Ge J. B. (2021). Oxidized low-density lipoprotein stimulates dendritic cells maturation via LOX-1-mediated MAPK/NF-κB pathway. Braz. J. Med. Biol. Res. 54, e11062. 10.1590/1414-431X2021e11062 34076144 PMC8186376

[B53] Huang X. Tang L. Wang F. Song G. (2014). Astragaloside IV attenuates allergic inflammation by regulation Th1/Th2 cytokine and enhancement CD4(+)CD25(+)Foxp3 T cells in ovalbumin-induced asthma. Immunobiology 219, 565–571. 10.1016/j.imbio.2014.03.005 24731407

[B54] Huang Z. Jiang K. Pi Y. Hou R. Liao Z. Cao Y. (2007). Molecular cloning and characterization of the yew gene encoding squalene synthase from Taxus cuspidata. J. Biochem. Mol. Biol. 40, 625–635. 10.5483/bmbrep.2007.40.5.625 17927893

[B55] Huo J. Y. Zou J. Ma H. H. Meng G. L. Huang Y. M. Yan X. L. (2024). Astragaloside IV microfibers assembling into injectable 3D-scaffolds with intrinsic immunoactivity for enhanced tumor vaccine efficacy. Chem. Eng. J. 498, 155511. 10.1016/j.cej.2024.155511

[B56] Ikram M. Jo M. H. Choe K. Khan A. Ahmad S. Saeed K. (2021). Cycloastragenol, a triterpenoid saponin, regulates oxidative stress, neurotrophic dysfunctions, neuroinflammation and apoptotic cell death in neurodegenerative conditions. Cells 10, 2719. 10.3390/cells10102719 34685699 PMC8534642

[B57] Ingersoll M. A. Platt A. M. Potteaux S. Randolph G. J. (2011). Monocyte trafficking in acute and chronic inflammation. Trends Immunol. 32, 470–477. 10.1016/j.it.2011.05.001 21664185 PMC3179572

[B58] Ivashkiv L. B. (2018). IFNγ: signalling, epigenetics and roles in immunity, metabolism, disease and cancer immunotherapy. Nat. Rev. Immunol. 18, 545–558. 10.1038/s41577-018-0029-z 29921905 PMC6340644

[B59] Jiang C. Zhou Z. Lin Y. Shan H. Xia W. Yin F. (2021). Astragaloside IV ameliorates steroid-induced osteonecrosis of the femoral head by repolarizing the phenotype of pro-inflammatory macrophages. Int. Immunopharmacol. 93, 107345. 10.1016/j.intimp.2020.107345 33563553

[B60] Jiang Z. Jiang J. X. Zhang G. X. (2014). Macrophages: a double-edged sword in experimental autoimmune encephalomyelitis. Immunol. Lett. 160, 17–22. 10.1016/j.imlet.2014.03.006 24698730 PMC6186449

[B61] Jiangbo Z. Xuying W. Yuping Z. Xili M. Yiwen Z. Tianbao Z. (2009). Effect of astragaloside IV on the embryo-fetal development of Sprague-Dawley rats and New Zealand white rabbits. J. Appl. Toxicol. 29, 381–385. 10.1002/jat.1422 19367606

[B62] Karin M. (2006). NF-kappaB and cancer: mechanisms and targets. Mol. Carcinog. 45, 355–361. 10.1002/mc.20217 16673382

[B63] Karnati H. K. Pasupuleti S. R. Kandi R. Undi R. B. Sahu I. Kannaki T. R. (2015). TLR-4 signalling pathway: MyD88 independent pathway up-regulation in chicken breeds upon LPS treatment. Vet. Res. Commun. 39, 73–78. 10.1007/s11259-014-9621-2 25417198

[B64] Kim Y. B. Thwe A. A. Li X. Tuan P. A. Lee S. Lee J. W. (2014). Accumulation of astragalosides and related gene expression in different organs of Astragalus membranaceus Bge. var mongholicus (Bge.). Molecules 19, 10922–10935. 10.3390/molecules190810922 25068786 PMC6270750

[B65] Kitagawa I. Wang H. Yoshikawa M. Takagi A. (1983). Saponin and sapogenol. XXXV. Chemical constituents of astragali radix, the root of Astragalus membranaceus Bunge. (2). Astragalosides I, II and IV, acetylastragaloside I and isoastragalosides I and II. Chem. Pharm. Bull. 31, 698–708. 10.1248/cpb.31.698

[B66] Kollewe C. Mackensen A. C. Neumann D. Knop J. Cao P. Li S. (2004). Sequential autophosphorylation steps in the interleukin-1 receptor-associated kinase-1 regulate its availability as an adapter in interleukin-1 signaling. J. Biol. Chem. 279, 5227–5236. 10.1074/jbc.M309251200 14625308

[B67] Kong C. F. Sun J. R. Hu X. Z. Li G. Z. Wu S. (2024). A tumor targeted nano micelle carrying astragaloside IV for combination treatment of bladder cancer. Sci. Rep. 14, 17704. 10.1038/s41598-024-66010-3 39085255 PMC11291986

[B68] Kuang G. Zhao Y. Wang L. Wen T. Liu P. Ma B. (2024). Astragaloside IV alleviates acute hepatic injury by regulating macrophage polarization and pyroptosis via activation of the AMPK/SIRT1 signaling pathway. Phytother. Res. 39, 733–746. 10.1002/ptr.8403 39660635

[B69] Kundu P. Ling T. W. Korecka A. Li Y. D'Arienzo R. Bunte R. M. (2014). Absence of intestinal PPARγ aggravates acute infectious colitis in mice through a lipocalin-2-dependent pathway. PLoS Pathog. 10, e1003887. 10.1371/journal.ppat.1003887 24465207 PMC3900641

[B70] Laplante M. Sabatini D. M. (2012). mTOR signaling in growth control and disease. Cell 149, 274–293. 10.1016/j.cell.2012.03.017 22500797 PMC3331679

[B71] Leslie K. A. Russell M. A. Taniguchi K. Richardson S. J. Morgan N. G. (2019). The transcription factor STAT6 plays a critical role in promoting beta cell viability and is depleted in islets of individuals with type 1 diabetes. Diabetologia 62, 87–98. 10.1007/s00125-018-4750-8 30338340 PMC6290857

[B72] Levy M. M. Fink M. P. Marshall J. C. Abraham E. Angus D. Cook D. (2003). 2001 SCCM/ESICM/ACCP/ATS/SIS international sepsis definitions conference. Intensive Care Med. 29, 530–538. 10.1007/s00134-003-1662-x 12664219

[B73] Li C. Deng C. Wang S. Dong X. Dai B. Guo W. (2024a). A novel role for the ROS-ATM-Chk2 axis mediated metabolic and cell cycle reprogramming in the M1 macrophage polarization. Redox Biol. 70, 103059. 10.1016/j.redox.2024.103059 38316066 PMC10862067

[B74] Li C. J. Xiao Y. Sun Y. C. He W. Z. Liu L. Huang M. (2022). Senescent immune cells release grancalcin to promote skeletal aging. Cell. Metab. 34, 184–185. 10.1016/j.cmet.2021.12.003 34986333

[B75] Li H. Y. Wan H. Y. Xia T. Chen M. H. Zhang Y. Luo X. M. (2015). Therapeutic angiogenesis in ischemic muscles after local injection of fragmented fibers with loaded traditional Chinese medicine. Nanoscale 7, 13075–13087. 10.1039/c5nr02005k 26176198

[B76] Li J. J. Li Y. L. Chu W. Li G. Q. Zhang M. Dong J. J. (2023a). Astragaloside IV alleviates cytarabine-induced intestinal mucositis by remodeling macrophage polarization through AKT signaling. Phytomedicine 109, 154605. 10.1016/j.phymed.2022.154605 36610133

[B77] Li L. Gan H. Jin H. Fang Y. Yang Y. Zhang J. (2021a). Astragaloside IV promotes microglia/macrophages M2 polarization and enhances neurogenesis and angiogenesis through PPARγ pathway after cerebral ischemia/reperfusion injury in rats. Int. Immunopharmacol. 92, 107335. 10.1016/j.intimp.2020.107335 33429332

[B78] Li L. Zou J. Zhou M. Li H. Zhou T. Liu X. (2024b). Phenylsulfate-induced oxidative stress and mitochondrial dysfunction in podocytes are ameliorated by Astragaloside IV activation of the SIRT1/PGC1α/Nrf1 signaling pathway. Biomed. Pharmacother. 177, 117008. 10.1016/j.biopha.2024.117008 38901196

[B79] Li M. Niu Y. Tian L. Zhang T. Zhou S. Wang L. (2024c). Astragaloside IV alleviates macrophage senescence and d-galactose-induced bone loss in mice through STING/NF-κB pathway. Int. Immunopharmacol. 129, 111588. 10.1016/j.intimp.2024.111588 38290207

[B80] Li M. Wang X. Wang Y. Bao S. Chang Q. Liu L. (2021b). Strategies for remodeling the tumor microenvironment using active ingredients of ginseng—a promising approach for cancer therapy. Front. Pharmacol. 12, 797634. 10.3389/fphar.2021.797634 35002732 PMC8727883

[B81] Li Q. Wu T. Zhao L. Pei J. Wang Z. Xiao W. (2019). Highly efficient biotransformation of astragaloside IV to cycloastragenol by sugar-stimulated β-glucosidase and β-xylosidase from Dictyoglomus thermophilum. J. Microbiol. Biotechnol. 29, 1882–1893. 10.4014/jmb.1807.07020 30176709

[B82] Li X. Kempf S. Günther S. Hu J. Fleming I. (2023b). 11,12-EET regulates PPAR-γ expression to modulate TGF-β-mediated macrophage polarization. Cells 12, 700. 10.3390/cells12050700 36899838 PMC10000544

[B83] Lin J. Pan X. Huang C. Gu M. Chen X. Zheng X. (2020). Dual regulation of microglia and neurons by astragaloside IV-mediated mTORC1 suppression promotes functional recovery after acute spinal cord injury. J. Cell. Mol. Med. 24, 671–685. 10.1111/jcmm.14776 31675186 PMC6933381

[B84] Linton M. F. Moslehi J. J. Babaev V. R. (2019). Akt signaling in macrophage polarization, survival, and atherosclerosis. Int. J. Mol. Sci. 20, 2703. 10.3390/ijms20112703 31159424 PMC6600269

[B85] Litterst C. M. Pfitzner E. (2001). Transcriptional activation by STAT6 requires the direct interaction with NCoA-1. J. Biol. Chem. 276, 45713–45721. 10.1074/jbc.M108132200 11574547

[B86] Liu F. Qiu H. Xue M. Zhang S. Zhang X. Xu J. (2019). MSC-secreted TGF-β regulates lipopolysaccharide-stimulated macrophage M2-like polarization via the Akt/FoxO1 pathway. Stem Cell. Res. Ther. 10, 345. 10.1186/s13287-019-1447-y 31771622 PMC6878630

[B87] Liu F. Ran F. He H. Q. Chen L. Y. (2020). Astragaloside IV exerts anti-tumor effect on murine colorectal cancer by re-educating tumor-associated macrophage. Arch. Immunol. Ther. Exp. 68, 33. 10.1007/s00005-020-00598-y 33095374

[B88] Liu K. Wang H. Zhou J. M. Zhu S. Y. Ma M. Xiao H. (2024). HMGB1 in exosomes derived from gastric cancer cells induces M2-like macrophage polarization by inhibiting the NF-κB signaling pathway. Cell. Biol. Int. 48, 334–346. 10.1002/cbin.12110 38105539

[B89] Liu K. Zhao E. Ilyas G. Lalazar G. Lin Y. Haseeb M. (2015). Impaired macrophage autophagy increases the immune response in obese mice by promoting proinflammatory macrophage polarization. Autophagy 11, 271–284. 10.1080/15548627.2015.1009787 25650776 PMC4502775

[B90] Liu L. C. Wang W. F. Hong W. H. Jin Y. Y. Wang L. C. Liu S. J. (2022). Photothermal 2D nanosheets combined with astragaloside IV for antibacterial properties and promoting angiogenesis to treat infected wounds. Front. Bioeng. Biotechnol. 9, 826011. 10.3389/fbioe.2021.826011 35223823 PMC8864217

[B91] Liu R. Jiang H. Tian Y. Zhao W. Wu X. (2016). Astragaloside IV protects against polymicrobial sepsis through inhibiting inflammatory response and apoptosis of lymphocytes. J. Surg. Res. 200, 315–323. 10.1016/j.jss.2015.08.024 26375505

[B92] Liu T. Wang L. Liang P. Wang X. Liu Y. Cai J. (2021a). USP19 suppresses inflammation and promotes M2-like macrophage polarization by manipulating NLRP3 function via autophagy. Cell. Mol. Immunol. 18, 2431–2442. 10.1038/s41423-020-00567-7 33097834 PMC8484569

[B93] Liu X. Lu Y. Qin S. (2021b). Atezolizumab and bevacizumab for hepatocellular carcinoma: mechanism, pharmacokinetics and future treatment strategies. Future Oncol. 17, 2243–2256. 10.2217/fon-2020-1290 33663220

[B94] Lowes M. A. Suárez-Fariñas M. Krueger J. G. (2014). Immunology of psoriasis. Annu. Rev. Immunol. 32, 227–255. 10.1146/annurev-immunol-032713-120225 24655295 PMC4229247

[B95] Luo W. Xu Q. Wang Q. Wu H. Hua J. (2017). Effect of modulation of PPAR-γ activity on Kupffer cells M1/M2 polarization in the development of non-alcoholic fatty liver disease. Sci. Rep. 7, 44612. 10.1038/srep44612 28300213 PMC5353732

[B96] Luo X. Huang P. Yuan B. Liu T. Lan F. Lu X. (2016). Astragaloside IV enhances diabetic wound healing involving upregulation of alternatively activated macrophages. Int. Immunopharmacol. 35, 22–28. 10.1016/j.intimp.2016.03.020 27016716

[B97] Luyendyk J. P. Schabbauer G. A. Tencati M. Holscher T. Pawlinski R. Mackman N. (2008). Genetic analysis of the role of the PI3K-Akt pathway in lipopolysaccharide-induced cytokine and tissue factor gene expression in monocytes/macrophages. J. Immunol. 180, 4218–4226. 10.4049/jimmunol.180.6.4218 18322234 PMC2834303

[B98] Ma P. K. Wei B. H. Cao Y. L. Miao Q. Chen N. Guo C. E. (2017). Pharmacokinetics, metabolism, and excretion of cycloastragenol, a potent telomerase activator in rats. Xenobiotica 47, 526–537. 10.1080/00498254.2016.1204568 27412909

[B99] Magedans Y. V. D. Phillips M. A. Fett-Neto A. G. (2021). Production of plant bioactive triterpenoid saponins: from metabolites to genes and back. Phytochem. Rev. 20, 461–482. 10.1007/s11101-020-09722-4

[B100] Manguso R. T. Pope H. W. Zimmer M. D. Brown F. D. Yates K. B. Miller B. C. (2017). *In vivo* CRISPR screening identifies Ptpn2 as a cancer immunotherapy target. Nature 547, 413–418. 10.1038/nature23270 28723893 PMC5924693

[B101] Martinez F. O. Sica A. Mantovani A. Locati M. (2008). Macrophage activation and polarization. Front. Biosci. 13, 453–461. 10.2741/2692 17981560

[B102] Min L. Wang H. Qi H. (2022). Astragaloside IV inhibits the progression of liver cancer by modulating macrophage polarization through the TLR4/NF-κB/STAT3 signaling pathway. Am. J. Transl. Res. 14, 1551–1566. 35422920 PMC8991133

[B103] Murdoch C. Giannoudis A. Lewis C. E. (2004). Mechanisms regulating the recruitment of macrophages into hypoxic areas of tumors and other ischemic tissues. Blood 104, 2224–2234. 10.1182/blood-2004-03-1109 15231578

[B104] Nakagawa Y. Chiba K. (2015). Diversity and plasticity of microglial cells in psychiatric and neurological disorders. Pharmacol. Ther. 154, 21–35. 10.1016/j.pharmthera.2015.06.010 26129625

[B105] Nourshargh S. Alon R. (2014). Leukocyte migration into inflamed tissues. Immunity 41, 694–707. 10.1016/j.immuni.2014.10.008 25517612

[B106] O’Shea J. J. Schwartz D. M. Villarino A. V. Gadina M. McInnes I. B. Laurence A. (2015). The JAK-STAT pathway: impact on human disease and therapeutic intervention. Annu. Rev. Med. 66, 311–328. 10.1146/annurev-med-051113-024537 25587654 PMC5634336

[B107] Peng X. R. Zhang T. T. Wu Y. J. Wang X. Y. Liu R. Jin X. (2023). mPEG-CS-modified flexible liposomes-reinforced thermosensitive sol-gel reversible hydrogels for ocular delivery of multiple drugs with enhanced synergism. Colloids Surf. B Biointerfaces 231, 113560. 10.1016/j.colsurfb.2023.113560 37812861

[B108] Piccolo V. Curina A. Genua M. Ghisletti S. Simonatto M. Sabò A. (2017). Opposing macrophage polarization programs show extensive epigenomic and transcriptional cross-talk. Nat. Immunol. 18, 530–540. 10.1038/ni.3710 28288101 PMC5524187

[B109] Qing L. S. Chen T. B. Sun W. X. Chen L. Luo P. Zhang Z. F. (2019). Pharmacokinetics comparison, intestinal absorption and acute toxicity assessment of a novel water-soluble astragaloside IV derivative (astragalosidic acid, LS-102). Eur. J. Drug Metab. Pharmacokinet. 44, 251–259. 10.1007/s13318-018-0515-5 30315409

[B110] Qu T. Suo S. Yan L. Wang L. Liang T. (2025). Anti-inflammatory effects of astragaloside IV-chitosan nanoparticles and associated anti-inflammatory mechanisms based on liquid chromatography-mass spectrometry metabolomic analysis of RAW264.7 cells. Chem. Biodivers., e202402234. 10.1002/cbdv.202402234 39891602

[B111] Que Y. D. Yang Y. H. Zafar H. Wang D. M. (2022). Tetracycline-grafted mPEG-PLGA micelles for bone-targeting and osteoporotic improvement. Front. Pharmacol. 13, 993095. 10.3389/fphar.2022.993095 36188546 PMC9515468

[B112] Raftery N. Stevenson N. J. (2017). Advances in anti-viral immune defence: revealing the importance of the IFN JAK/STAT pathway. Cell. Mol. Life Sci. 74, 2525–2535. 10.1007/s00018-017-2520-2 28432378 PMC7079803

[B113] Rayees S. Rochford I. Joshi J. C. Joshi B. Banerjee S. Mehta D. (2020). Macrophage TLR4 and PAR2 signaling: role in regulating vascular inflammatory injury and repair. Front. Immunol. 11, 2091. 10.3389/fimmu.2020.02091 33072072 PMC7530636

[B114] Raza F. Jiang L. D. Zhang S. L. Zafar H. Qiu Y. J. Su J. (2024). SP94 engineered erythrocyte membrane enhanced the targeted delivery of biomimetic nanosuspension with Ido immunotherapy and chemotherapy in liver cancer. Front. Immunol. 491, 151709. 10.1016/j.cej.2024.151709

[B115] Ren C. Zhao X. Liu K. Wang L. Chen Q. Jiang H. (2023). Research progress of natural medicine Astragalus mongholicus Bunge in treatment of myocardial fibrosis. J. Ethnopharmacol. 305, 116128. 10.1016/j.jep.2022.116128 36623754

[B116] Rios J. L. Waterman P. G. (1997). A review of the pharmacology and toxicology of Astragalus. Phytother. Res. 11, 411–418. 10.1002/(SICI)1099-1573(199709)11:6<411::AID-PTR132>3.0.CO;2-6

[B117] Russell F. M. Hardie D. G. (2020). AMP-activated protein kinase: do we need activators or inhibitors to treat or prevent cancer? Int. J. Mol. Sci. 22, 186. 10.3390/ijms22010186 33375416 PMC7795930

[B118] Sag D. Carling D. Stout R. D. Suttles J. (2008). Adenosine 5’-monophosphate-activated protein kinase promotes macrophage polarization to an anti-inflammatory functional phenotype. J. Immunol. 181, 8633–8641. 10.4049/jimmunol.181.12.8633 19050283 PMC2756051

[B119] Sedighzadeh S. S. Khoshbin A. P. Razi S. Keshavarz-Fathi M. Rezaei N. (2021). A narrative review of tumor-associated macrophages in lung cancer: regulation of macrophage polarization and therapeutic implications. Transl. Lung Cancer Res. 10, 1889–1916. 10.21037/tlcr-20-1241 34012800 PMC8107755

[B120] Sehnert B. Burkhardt H. Dübel S. Voll R. E. (2020). Cell-type targeted NF-κB inhibition for the treatment of inflammatory diseases. Cells 9, 1627. 10.3390/cells9071627 32640727 PMC7407293

[B121] Shan Y. Yu M. Dai H. Zhu X. Wang F. You Y. (2024). The role of macrophage-derived exosomes in reversing peritoneal fibrosis: insights from astragaloside IV. Phytomedicine 129, 155683. 10.1016/j.phymed.2024.155683 38701543

[B122] Shen L. Li Y. Hu G. Song X. Wang X. Li X. (2023). Astragaloside IV suppresses the migration and EMT progression of cervical cancer cells by inhibiting macrophage M2 polarization through TGFβ/Smad2/3 signaling. Funct. Integr. Genomics 23, 133. 10.1007/s10142-023-01017-z 37081108

[B123] Shih R. H. Wang C. Y. Yang C. M. (2015). NF-kappaB signaling pathways in neurological inflammation: a mini review. Front. Mol. Neurosci. 8, 77. 10.3389/fnmol.2015.00077 26733801 PMC4683208

[B124] Sica A. Bronte V. (2007). Altered macrophage differentiation and immune dysfunction in tumor development. J. Clin. Invest. 117, 1155–1166. 10.1172/JCI31422 17476345 PMC1857267

[B125] Sica A. Mantovani A. (2012). Macrophage plasticity and polarization: *in vivo* veritas. J. Clin. Invest. 122, 787–795. 10.1172/JCI59643 22378047 PMC3287223

[B126] Simsek E. Yildirim K. Akcit E. T. Atas C. Kocak O. Altinkaynak A. (2024). The *in vitro* evaluation of *in silico*-designed synthetic peptides AKVUAM-1 and AKVUAM-2 on human lung fibroblast cell line MRC5 and *Mycobacterium tuberculosis* isolates. Microb. Pathog. 197, 107027. 10.1016/j.micpath.2024.107027 39426636

[B127] Song G. Ouyang G. Bao S. (2005). The activation of Akt/PKB signaling pathway and cell survival. J. Cell. Mol. Med. 9, 59–71. 10.1111/j.1582-4934.2005.tb00337.x 15784165 PMC6741304

[B128] Song S. Li Y. Liu X. Yu J. Li Z. Liang K. (2023). Study on the biotransformation and activities of astragalosides from Astragali Radix *in vitro* and *in vivo* . J. Agric. Food Chem. 71, 17924–17946. 10.1021/acs.jafc.3c05405 37940610

[B129] Song Y. D. Li X. Z. Liu F. F. Zhu H. B. Shen Y. (2021). Isoalantolactone alleviates ovalbumin-induced asthmatic inflammation by reducing alternatively activated macrophage and STAT6/PPAR-γ/KLF4 signals. Mol. Med. Rep. 24, 701. 10.3892/mmr.2021.12340 34368878

[B130] Steinberg G. R. Hardie D. G. (2023). New insights into activation and function of the AMPK. Nat. Rev. Mol. Cell. Biol. 24, 255–272. 10.1038/s41580-022-00547-x 36316383

[B131] Sun K. Jing X. Guo J. Yao X. Guo F. (2021). Mitophagy in degenerative joint diseases. Autophagy 17, 2082–2092. 10.1080/15548627.2020.1822097 32967533 PMC8496714

[B132] Sun R. Zhang A. Ge Y. Gou J. Yin T. He H. (2020). Ultra-small-size astragaloside-IV loaded lipid nanocapsules eye drops for the effective management of dry age-related macular degeneration. Expert Opin. Drug Deliv. 17, 1305–1320. 10.1080/17425247.2020.1783236 32538226

[B133] Szabo N. J. (2014). Dietary safety of cycloastragenol from Astragalus spp.: subchronic toxicity and genotoxicity studies. Food Chem. Toxicol. 64, 322–334. 10.1016/j.fct.2013.11.041 24316212

[B134] Szewczuk M. Boguszewska K. Kaźmierczak-Barańska J. Karwowski B. T. (2020). The role of AMPK in metabolism and its influence on DNA damage repair. Mol. Biol. Rep. 47, 9075–9086. 10.1007/s11033-020-05900-x 33070285 PMC7674386

[B135] Takeuchi D. M. Kishino S. Ozeki Y. Fukami H. Ogawa J. (2022). Analysis of astragaloside IV metabolism to cycloastragenol in human gut microorganism, bifidobacteria, and lactic acid bacteria. Biosci. Biotechnol. Biochem. 86, 1467–1475. 10.1093/bbb/zbac130 35904311

[B136] Tang L. Zhu M. Che X. Yang X. Xu Y. Ma Q. (2022a). Astragaloside IV targets macrophages to alleviate renal ischemia-reperfusion injury via the crosstalk between Hif-1α and NF-κB (p65)/Smad7 pathways. J. Pers. Med. 13, 59. 10.3390/jpm13010059 36675720 PMC9863138

[B137] Tang L. M. Li X. Qin Y. Geng X. Y. Wang R. W. Tan W. H. (2022b). The construction of oligonucleotide-cycloastragenol and the renoprotective effect study. Front. Bioeng. Biotechnol. 10, 1027517. 10.3389/fbioe.2022.1027517 36518194 PMC9742277

[B138] Tian L. Zhao J. L. Kang J. Q. Guo S. B. Zhang N. Shang L. (2021). Astragaloside IV alleviates the experimental DSS-induced colitis by remodeling macrophage polarization through STAT signaling. Front. Immunol. 12, 740565. 10.3389/fimmu.2021.740565 34589089 PMC8473681

[B139] U.S. Food and Drug Administration (n.d.). NDC search results for telomere finished products. FDA Drug Registration Listing System. Available online at: https://dps.fda.gov/ndc/searchresult?selection=finished_product&content=PROPRIETARYNAME&type=Telomere (Accessed April 17, 2025).

[B140] Vergadi E. Ieronymaki E. Lyroni K. Vaporidi K. Tsatsanis C. (2017). Akt signaling pathway in macrophage activation and M1/M2 polarization. J. Immunol. 198, 1006–1014. 10.4049/jimmunol.1601515 28115590

[B141] Walsh M. C. Lee J. Choi Y. (2015). Tumor necrosis factor receptor-associated factor 6 (TRAF6) regulation of development, function, and homeostasis of the immune system. Immunol. Rev. 266, 72–92. 10.1111/imr.12302 26085208 PMC4799835

[B142] Wan Y. Xu L. Wang Y. Tuerdi N. Ye M. Qi R. (2018). Preventive effects of astragaloside IV and its active sapogenin cycloastragenol on cardiac fibrosis of mice by inhibiting the NLRP3 inflammasome. Eur. J. Pharmacol. 833, 545–554. 10.1016/j.ejphar.2018.06.016 29913124

[B143] Wang B. Chen M. Z. (2014). Astragaloside IV possesses antiarthritic effect by preventing interleukin 1β-induced joint inflammation and cartilage damage. Arch. Pharm. Res. 37, 793–802. 10.1007/s12272-014-0336-2 24469603

[B144] Wang C. Y. Li Y. Hao M. J. Li W. M. (2018a). Astragaloside IV inhibits triglyceride accumulation in insulin-resistant HepG2 cells via AMPK-induced SREBP-1c phosphorylation. Front. Pharmacol. 9, 345. 10.3389/fphar.2018.00345 29713279 PMC5911465

[B145] Wang H. Peters T. Sindrilaru A. Scharffetter-Kochanek K. (2009a). Key role of macrophages in the pathogenesis of CD18 hypomorphic murine model of psoriasis. J. Invest. Dermatol. 129, 1100–1114. 10.1038/jid.2009.43 19242511

[B146] Wang H. Tian T. Zhang J. (2021a). Tumor-associated macrophages (TAMs) in colorectal cancer (CRC): from mechanism to therapy and prognosis. Int. J. Mol. Sci. 22, 8470. 10.3390/ijms22168470 34445193 PMC8395168

[B147] Wang J. Ma A. Zhao M. Zhu H. (2017). AMPK activation reduces the number of atheromata macrophages in ApoE deficient mice. Atherosclerosis 258, 97–107. 10.1016/j.atherosclerosis.2017.01.036 28235712

[B148] Wang J. Wang Y. Chu Y. Li Z. Yu X. Huang Z. (2021b). Tumor-derived adenosine promotes macrophage proliferation in human hepatocellular carcinoma. J. Hepatol. 74, 627–637. 10.1016/j.jhep.2020.10.021 33137360

[B149] Wang J. Zhou Y. Wu S. Huang K. Thapa S. Tao L. (2018b). Astragaloside IV attenuated 3,4-Benzopyrene-induced abdominal aortic aneurysm by ameliorating macrophage-mediated inflammation. Front. Pharmacol. 9, 496. 10.3389/fphar.2018.00496 29872394 PMC5972279

[B150] Wang L. Chen Y. (2017). Efficient biotransformation of astragaloside IV to cycloastragenol by Bacillus sp. LG-502. Appl. Biochem. Biotechnol. 183, 1488–1502. 10.1007/s12010-017-2517-1 28593602

[B151] Wang N. Zhang X. Ma Z. Niu J. Ma S. Wenjie W. (2020). Combination of tanshinone IIA and astragaloside IV attenuate atherosclerotic plaque vulnerability in ApoE^-^/^-^ mice by activating PI3K/AKT signaling and suppressing TRL4/NF-κB signaling. Biomed. Pharmacother. 123, 109729. 10.1016/j.biopha.2019.109729 31887543

[B152] Wang S. Li J. Huang H. Gao W. Zhuang C. Li B. (2009b). Anti-hepatitis B virus activities of astragaloside IV isolated from Radix Astragali. Biol. Pharm. Bull. 32, 132–135. 10.1248/bpb.32.132 19122295

[B153] Wang X. Gao S. Song L. Liu M. Sun Z. Liu J. (2021c). Astragaloside IV antagonizes M2 phenotype macrophage polarization-evoked ovarian cancer cell malignant progression by suppressing the HMGB1-TLR4 axis. Mol. Immunol. 130, 113–121. 10.1016/j.molimm.2020.11.014 33308900

[B154] Wang X. R. Luan J. X. Guo Z. A. (2024). Mechanism of astragaloside IV in treatment of renal tubulointerstitial fibrosis. Chin. J. Integr. Med. 31, 474–480. 10.1007/s11655-024-3805-6 38850482

[B155] Wu S. Zhou M. Zhou H. Han L. Liu H. (2025). Astragaloside IV- loaded biomimetic nanoparticles target IκBα to regulate neutrophil extracellular trap formation for sepsis therapy. J. Nanobiotechnol. 23, 155. 10.1186/s12951-025-03260-x PMC11869569 40022068

[B156] Wu T. Wu S. Gao H. Liu H. Feng J. Yin G. (2024). Astragaloside IV augments anti-PD-1 therapy to suppress tumor growth in lung cancer by remodeling the tumor microenvironment. Eur. J. Histochem. 68, 4098. 10.4081/ejh.2024.4098 39440587 PMC11558310

[B157] Xu B. Huang J. P. Peng G. Cao W. Liu Z. Chen Y. (2024). Total biosynthesis of the medicinal triterpenoid saponin astragalosides. Nat. Plants 10, 1826–1837. 10.1038/s41477-024-01827-4 39433972

[B158] Xu F. Cui W. Q. Wei Y. Cui J. Qiu J. Hu L. L. (2018). Astragaloside IV inhibits lung cancer progression and metastasis by modulating macrophage polarization through AMPK signaling. J. Exp. Clin. Cancer Res. 37, 207. 10.1186/s13046-018-0878-0 30157903 PMC6116548

[B159] Xu H. He Y. Chen S. Meng C. Liu Q. Huang X. J. (2025). Blocking the CCL5/CCL7-CCR1 axis regulates macrophage polarization through NF-κB pathway to alleviate the progression of osteoarthritis. Int. Immunopharmacol. 147, 114027. 10.1016/j.intimp.2025.114027 39805173

[B160] Xu M. Yin J. Xie L. Zhang J. Zou C. Zou J. (2013). Pharmacokinetics and tolerance of toal astragalosides after intravenous infusion of astragalosides injection in healthy Chinese volunteers. Phytomedicine 20, 1105–1111. 10.1016/j.phymed.2013.05.004 23838148

[B161] Xuying W. Jiangbo Z. Yuping Z. Xili M. Yiwen Z. Tianbao Z. (2010). Effect of astragaloside IV on the general and peripartum reproductive toxicity in Sprague-Dawley rats. Int. J. Toxicol. 29, 505–516. 10.1177/1091581810376840 20884860

[B162] Yadav J. Tripathi T. Chaudhary A. Janjua D. Joshi U. Aggarwal N. (2025). Influence of head and neck cancer exosomes on macrophage polarization. Cytokine 186, 156831. 10.1016/j.cyto.2024.156831 39700664

[B163] Yang J. Jiang H. Wu C. Lin Y. Tan G. Zhan J. (2025). Copper silicate nanoparticle-mediated delivery of astragaloside-IV for osteoarthritis treatment by remodeling the articular cartilage microenvironment. J. Control. Release 381, 113583. 10.1016/j.jconrel.2025.113583 40032006

[B164] Yang T. Xie S. Cao L. Li M. Ding L. Wang L. (2024). Astragaloside IV modulates gut macrophages M1/M2 polarization by reshaping gut microbiota and short chain fatty acids in sepsis. Shock 61, 120–131. 10.1097/SHK.0000000000002262 37962207 PMC11841723

[B165] Yang X. F. Peng Y. R. Wang Y. E. Zheng Y. X. He Y. M. Pan J. L. (2023). Curcumae rhizoma exosomes-like nanoparticles loaded Astragalus components improve the absorption and enhance anti-tumor effect. J. Drug Deliv. Sci. Technol. 81, 104274. 10.1016/j.jddst.2023.104274

[B166] Yao J. Liu J. He Y. Liu L. Xu Z. Lin X. (2023). Systems pharmacology reveals the mechanism of Astragaloside IV in improving immune activity on cyclophosphamide-induced immunosuppressed mice. J. Ethnopharmacol. 313, 116533. 10.1016/j.jep.2023.116533 37100262

[B167] Yi X. W. Fransisca C. He Y. Liu Y. H. Lu S. He L. Y. (2017). Photodynamic effects on Fonsecaea monophora conidia and RAW264.7 *in vitro* . J. Photochem. Photobiol. B 176, 112–117. 10.1016/j.jphotobiol.2017.09.001 28992604

[B168] Ying Y. Sun C. B. Zhang S. Q. Chen B. J. Yu J. Z. Liu F. Y. (2021). Induction of autophagy via the TLR4/NF-κB signaling pathway by astragaloside IV contributes to the amelioration of inflammation in RAW264.7 cells. Biomed. Pharmacother. 137, 111271. 10.1016/j.biopha.2021.111271 33561643

[B169] Yu J. Mu B. Guo M. Liu C. Meng T. Yan Y. (2023a). Astragaloside IV inhibits experimental autoimmune encephalomyelitis by modulating the polarization of both microglia/macrophages and astrocytes. Folia Neuropathol. 61, 273–290. 10.5114/fn.2023.129066 37818688

[B170] Yu S. Y. Ouyang H. T. Yang J. Y. Huang X. L. Yang T. Duan J. P. (2007). Subchronic toxicity studies of Radix Astragali extract in rats and dogs. J. Ethnopharmacol. 110, 352–355. 10.1016/j.jep.2006.09.024 17052876

[B171] Yu Y. Hao J. Wang L. Zheng X. Xie C. Liu H. (2023b). Astragaloside IV antagonizes the malignant progression of breast cancer induced by macrophage M2 polarization through the TGF-β-regulated Akt/Foxo1 pathway. Pathol. Res. Pract. 249, 154766. 10.1016/j.prp.2023.154766 37633006

[B172] Yu Y. Zhou L. Yang Y. Liu Y. (2018). Cycloastragenol: an exciting novel candidate for age-associated diseases. Exp. Ther. Med. 16, 2175–2182. 10.3892/etm.2018.6501 30186456 PMC6122403

[B173] Zhang F. Wang H. Wang X. Jiang G. Liu H. Zhang G. (2016). TGF-β induces M2-like macrophage polarization via SNAIL-mediated suppression of a pro-inflammatory phenotype. Oncotarget 7, 52294–52306. 10.18632/oncotarget.10561 27418133 PMC5239552

[B174] Zhang M. Bao Y. O. Zhao C. X. Tian Y. G. Wang Z. L. Qiao X. (2024). A four-step biosynthetic pathway involving C-3 oxidation-reduction reactions from cycloastragenol to astragaloside IV in Astragalus membranaceus. Plant J. 120, 569–577. 10.1111/tpj.17001 39180339

[B175] Zhang M. He Y. Sun X. Li Q. Wang W. Zhao A. (2014). A high M1/M2 ratio of tumor-associated macrophages is associated with extended survival in ovarian cancer patients. J. Ovarian Res. 7, 19. 10.1186/1757-2215-7-19 24507759 PMC3939626

[B176] Zhang M. Yi Y. Gao B. H. Su H. F. Bao Y. O. Shi X. M. (2022a). Functional characterization and protein engineering of a triterpene 3-/6-/2'-O-glycosyltransferase reveal a conserved residue critical for the regiospecificity. Angew. Chem. Int. Ed. 61, e202113587. 10.1002/anie.202113587 34894044

[B177] Zhang Q. Shen C. Zhao N. Xu F. J. (2017). Redox-responsive and drug embedded silica nanoparticles with unique self-destruction features for efficient gene/drug codelivery. Adv. Funct. Mater. 27, 1606229. 10.1002/adfm.201606229

[B178] Zhang Q. Zhu L. L. Chen G. G. Du Y. (2007). Pharmacokinetics of astragaloside IV in beagle dogs. Eur. J. Drug Metab. Pharmacokinet. 32, 75–79. 10.1007/BF03190995 17702194

[B179] Zhang W. D. Zhang C. Liu R. H. Li H. L. Zhang J. T. Mao C. (2006). Preclinical pharmacokinetics and tissue distribution of a natural cardioprotective agent astragaloside IV in rats and dogs. Life Sci. 79, 808–815. 10.1016/j.lfs.2006.02.032 16564551

[B180] Zhang X. Qu H. Yang T. Liu Q. Zhou H. (2022b). Astragaloside IV attenuate MI-induced myocardial fibrosis and cardiac remodeling by inhibiting ROS/caspase-1/GSDMD signaling pathway. Cell Cycle 21, 2309–2322. 10.1080/15384101.2022.2093598 35770948 PMC9586672

[B181] Zhao X. Luo J. L. Huang Y. Mu L. Chen J. Y. Liang Z. (2023a). Injectable antiswelling and high-strength bioactive hydrogels with a wet adhesion and rapid gelling process to promote sutureless wound closure and scar-free repair of infectious wounds. ACS Nano 17, 22015–22034. 10.1021/acsnano.3c08625 37862553

[B182] Zhao X. Sun L. X. Wang J. Xu X. L. Ni S. T. Liu M. (2023b). Nose to brain delivery of astragaloside IV by β-asarone modified chitosan nanoparticles for multiple sclerosis therapy. Int. J. Pharm. 644, 123351. 10.1016/j.ijpharm.2023.123351 37640088

[B183] Zheng X. Weigert A. Reu S. Guenther S. Mansouri S. Bassaly B. (2020). Spatial density and distribution of tumor-associated macrophages predict survival in non-small cell lung carcinoma. Cancer Res. 80, 4414–4425. 10.1158/0008-5472.CAN-20-0069 32699134

[B184] Zhong Y. B. Liu W. J. Xiong Y. X. Li Y. M. Wan Q. Zhou W. (2022). Astragaloside IV alleviates ulcerative colitis by regulating the balance of Th17/Treg cells. Phytomedicine 104, 154287. 10.1016/j.phymed.2022.154287 35752072

[B185] Zhou D. Huang C. Lin Z. Zhan S. Kong L. Fang C. (2014). Macrophage polarization and function with emphasis on the evolving roles of coordinated regulation of cellular signaling pathways. Cell. Signal. 26, 192–197. 10.1016/j.cellsig.2013.11.004 24219909

[B186] Zhou J. Li L. Pu Y. Li H. Wu X. Wang Z. (2024). Astragaloside IV inhibits colorectal cancer metastasis by reducing extracellular vesicles release and suppressing M2-type TAMs activation. Heliyon 10, e31450. 10.1016/j.heliyon.2024.e31450 38831823 PMC11145472

[B187] Zhou J. Wang L. Peng C. Peng F. (2022). Co-targeting tumor angiogenesis and immunosuppressive tumor microenvironment: a perspective in ethnopharmacology. Front. Pharmacol. 13, 886198. 10.3389/fphar.2022.886198 35784750 PMC9242535

[B188] Zhou R. N. Song Y. L. Ruan J. Q. Wang Y. T. Yan R. (2012). Pharmacokinetic evidence on the contribution of intestinal bacterial conversion to beneficial effects of astragaloside IV, a marker compound of Astragali Radix, in traditional oral use of the herb. Drug Metab. Pharmacokinet. 27, 586–597. 10.2133/dmpk.dmpk-11-rg-160 22673033

[B189] Zhou S. Gu J. Liu R. Wei S. Wang Q. Shen H. (2018). Spermine alleviates acute liver injury by inhibiting liver-resident macrophage pro-inflammatory response through ATG5-dependent autophagy. Front. Immunol. 9, 948. 10.3389/fimmu.2018.00948 29770139 PMC5940752

[B190] Zhu J. Lee S. Ho M. K. C. Hu Y. Pang H. Ip F. C. F. (2010). *In vitro* intestinal absorption and first-pass intestinal and hepatic metabolism of cycloastragenol, a potent small molecule telomerase activator. Drug Metab. Pharmacokinet. 25, 477–486. 10.2133/dmpk.dmpk-10-rg-037 20877137

[B191] Zhu Y. P. Brown J. R. Sag D. Zhang L. Suttles J. (2015). Adenosine 5’-monophosphate-activated protein kinase regulates IL-10-mediated anti-inflammatory signaling pathways in macrophages. J. Immunol. 194, 584–594. 10.4049/jimmunol.1401024 25512602 PMC4343033

